# Mitochondria transfer in tissue homeostasis and diseases

**DOI:** 10.7150/ijbs.129709

**Published:** 2026-02-26

**Authors:** Lei Qin, Donghao Gan, Zecai Chen, Zhen Xu, Mingyue Wu, Bimin Gao, Yufeng Long, Xihong Mou, Wenqing Li, Weihong Yi, Guozhi Xiao

**Affiliations:** 1 Department of Orthopaedics, Shenzhen Nanshan People's Hospital, Affiliated Nanshan Hospital of Shenzhen University, Shenzhen, Guangdong Province, China.; 2 Department of Orthopaedics & Rehabilitation, Yale University School of Medicine, New Haven, USA.; 3 Department of Biochemistry, Homeostatic Medicine Institute, School of Medicine, Guangdong Provincial Key Laboratory of Cell Microenvironment and Disease Research, Shenzhen Key Laboratory of Cell Microenvironment, Southern University of Science and Technology, Shenzhen, Guangdong Province, China.

**Keywords:** vertical mitochondria transfer, horizontal mitochondria transfer, intercellular mitochondria transfer, tunneling nanotubes (TNT), extracellular vesicles (EVs), gap junctions (GJs)

## Abstract

Mitochondria serve as the essential powerhouse for virtually all eukaryotic cells and have been implicated in other crucial functions in both physiological and disease contexts. As cytoplasmic organelles, mitochondria are segregated and transported from parent to daughter cells during division or differentiation, a process known as vertical mitochondria transfer (VMT). A growing body of literature indicates that various cell types can export mitochondria for delivery to developmentally unrelated cell types without division, a process termed horizontal mitochondria transfer (HMT). In this review, we summarize current understanding of the modes of mitochondria transfer and illustrate the phenomenon of HMT across different tissue backgrounds, including the immune, cardiovascular, respiratory, hepatic, renal, musculoskeletal, adipose, and reproductive systems. Moreover, updated applications and functions of mitochondria transfer are discussed. Additionally, we also highlight the therapeutic potential of mitochondria transfer in current preclinical and clinical trials for inherited mitochondrial diseases, cancer, wound healing, and injuries of the respiratory and central nervous systems.

## Introduction

Mitochondria are essential double-membrane subcellular organelles that can be found in nearly all eukaryotic cells. Considering their important regulations in continuously supplying eukaryotes with energy in the form of adenosine triphosphate (ATP), mitochondria are often referred to as the “powerhouse of the cell”^1^. In addition to energy supply, these ubiquitously distributed intracellular organelles encompassing wide ranges of essential cellular functions in reactive oxygen species (ROS) control^2^, ^3^, calcium signaling and homeostasis^4^, and immune responses^5^. Furthermore, mitochondria can regulate the cellular state from normal to stress^6^, thus involve in cell death^7^, ^8^.

Mitochondria are unique organelles in cell. They undergo dynamic cycles of fission and fusion, enabling their functions and adaptations to metabolic changes or cellular stress during physical or pathological conditions^9^, ^10^. Moreover, they are the only organelles that harbor their own DNA (mtDNA), which consists 37 genes and is uniformly distributed within the mitochondrial matrix^11^. Mitochondrial proteomes encoded by mtDNA consist of more than 1000 proteins^12^, which include proteins that involved in a variety of important functions as protein synthesis, amino acid and nucleotide metabolism, fatty-acid catabolism, apoptosis, ion homeostasis and etc. Moreover, mitochondrial biogenesis enables a self-renewal route for mitochondrial repair and regeneration^12^. Importantly, the per-cell numbers, size, structure, distribution of mitochondria in individual cell are under active and dynamic modeling, which may vary depending on the cell type, cell cycle and environmental conditions. Studies show that the respiration rate and mitochondrial protein content are different among different species and tissues^13^. Moreover, the cristae structure also vary across the eukaryotic domain. The typical flat, lamellar cristae structure is commonly observed in most mammalian tissues, whereas tubular cristae have been seed in budding yeast such as *Saccharomyces cerevisiae* and discoidal cristae have been identified in protists such as *Trypanosoma*^14^.

As mitochondria located in the cytoplasm, when cells divide, parent cells pass mitochondria to daughter cells through a process called vertical inheritance or vertical mitochondria transfer (VMT)^15^. For the past twenty years, emerging evidence started to show that mitochondria can be transfer or delivered from donor cells to recipient cells between developmentally unrelated cell types. This process is called intercelluar or horizontal mitochondria transfer (HMT), which has been reported both *in vitro* conditions and *in vivo* systems. HMT can be achieved between the same types of cells or different types of cells, between the same types of tissues/organs or even in different systems, and between health or pathology-related circumstances. In this review, we introduce the origin of mitochondria, describe the mechanisms and functions of mitochondria transfer, and summarize updated understandings of HMT functions in different tissue backgrounds. We also highlight the current experimental and clinical trials of mitochondria transfer and discuss it as a new therapeutic potential in cancer, wound healing and other human diseases.

## The discovery, structure and evolution of mitochondria

The discovery of mitochondria was achieved by a German scientist Richard Altmann in around 1890^16^, ^17^, who developed a new approach for tissue fixation and staining. He observed strings of granules within nearly all cells he examined. He called this new structures “bioblasts” and proposed that these granules could be responsible for metabolic processes. Later on, another German scientist, Carl Benda published results with a different crystal violet staining and named the Richard Altmann's bioblasts as "mitochondrion" in 1898^16^. Mitochondrion is from the Greek words *mitos* (meaning thread) and *chondros* (meaning granule) with the plural being mitochondria. In 1900, Leonor Michaelis published his findings with dye Janus green stained mitochondria in living cells, proving that they were real and dynamic organelles in cells^16^, ^18^.

**Figure 1:**
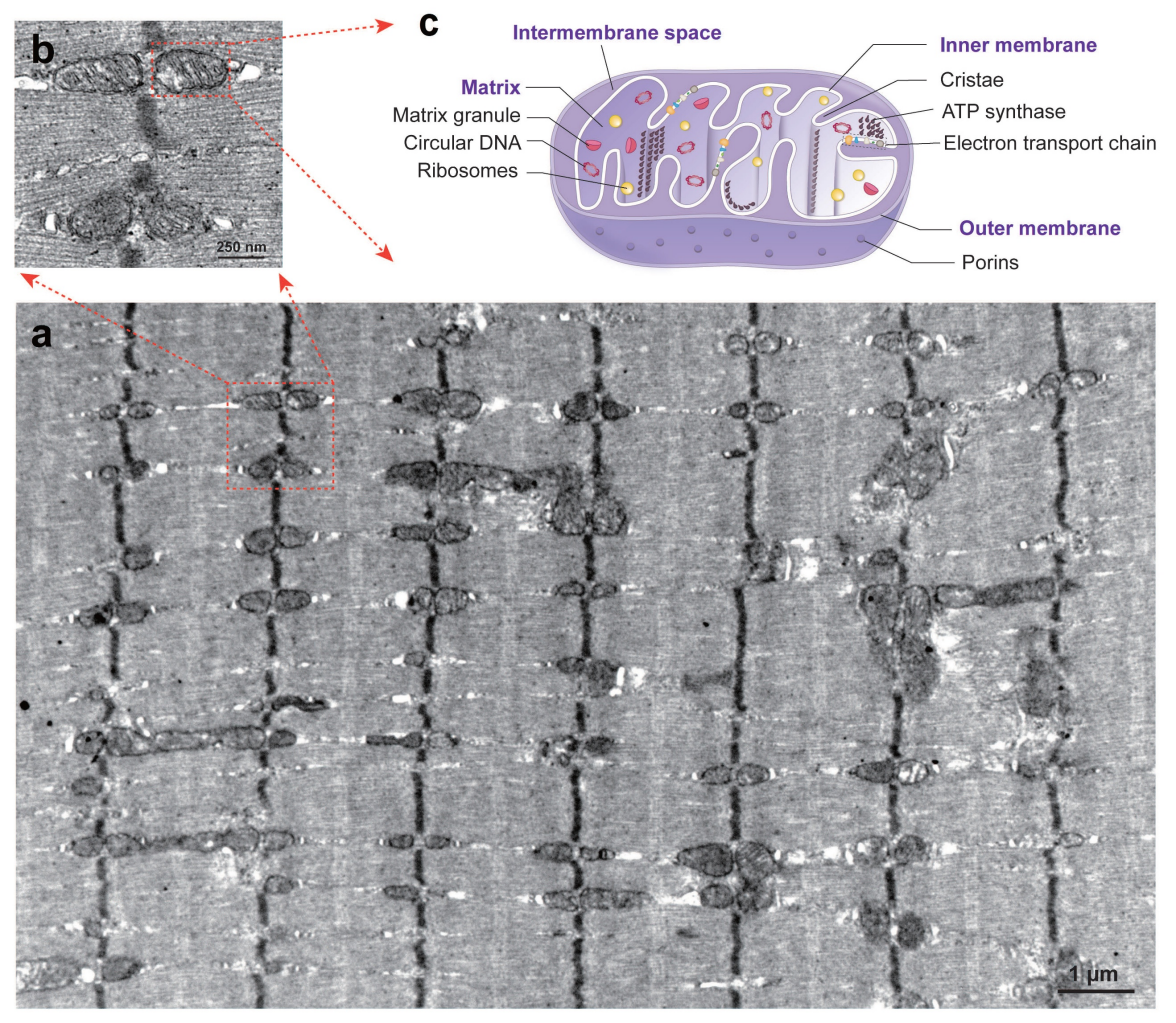
** Typical mitochondrial morphology in mouse skeletal muscles.** The typical mitochondrial morphology is illustrated with transmission electron microscopy (TEM) images detected from the cross-section of sole muscles from 4-month-old adult male mouse** (a, b)** and cartoon image for the inner structures of single mitochondrion** (c)**.

Mitochondria are ubiquitous organelles found in almost all eukaryotic cells, ranging from single-celled organisms and plants to vertebrates, including human being^19^, ^20^. Eukaryotic cell commonly has multiple mitochondria per cell, from several in single somatic cell to hundreds or thousands in multi-nuclear cells or mature oocytes^21^. Among all the tissues, skeletal muscle cells contain the greatest number of mitochondria due to the fused mucltinucleated myofibers and their high energy demands for contraction (***Figure 1. a***). The typical structure of a mitochondrion is a round to oval-shaped organelle with an average size of 0.75-3 μm^2^, ^22^. Single mitochondrion includes four essential parts (***Figure 1. b, c***): outer membrane, inner membrane, the intermembrane space between outer and inner membranes, and the matrix inside a mitochondrion.

The two membranes of mitochondria are largely different in nature: i.e., the outer membrane is lipid-rich and contains a rather low number of proteins, whereas the inner membrane contains hundreds of different integral membrane proteins^23^. The outer membrane contains multiple voltage-dependent anion channels (VDAC) called porins to control membrane permeability in mediating ion exchanges and metabolites between mitochondria and the cytosol. There are three isoforms of VDAC have been identified in mammals with different functions: VDAC1 is an important mediator for mitochondria-driven apoptosis; while VDAC2, conversely, serves an anti-apoptotic function; VDAC3 participates in the regulation of ROS production and mitochondrial quality control^24^. Another mitochondrial translocase of the outer membrane (TOM) complex mediates retrotranslocation of damaged mitochondrial proteins across the outer membrane in the mitochondria-associated degradation pathway (MAD) pathway for mitochondrial protein quality control^25^. Compared to the outer membrane, the inner membrane of mitochondria has been reported with a protein-to-lipid ratio of about 4 : 1, accounting for one of the most protein-rich membranes of the eukaryotic cell^26^. The inner membrane forms folds known as cristae. This peculiar membrane architecture is essential for diverse mitochondrial functions, including oxidative phosphorylation and the biosynthesis of cellular building blocks^27^. During the cristae formation, the mitochondrial contact site and cristae-organizing system (MICOS) synthesize key mitochondrial phospholipids which cooperate with cristae-shaping proteins and contribute to establish the inner membrane architecture^14^. The cristae increases the surface area of inner membrane and make it available for ATP productions through the electron transport chain and other chemical reactions^28^. The intermembrane space is the most constricted sub-mitochondrial compartment, which hosts only around 5% of the mitochondrial proteome and is involved in the regulation of ion concentrations^29^. Matrix is the innermost compartment of a mitochondrion, which contains mtDNA, ribosomes, various metabolic enzymes for oxidative phosphorylation. Two major proteases of the mitochondrial matrix, LON and ClpXP, have been recognized with essential functions in mitochondrial protein quality control, mitochondrial gene expression and respiratory chain function maintenance^30^.

The double membranes and unique genome of mitochondria distinct them from other intracellular organelles, which indicates the special origin of mitochondria during evolution. Several features of mitochondria are similar to bacteria. For instance, their circular genomic DNA are characterized by hypomethylated CpG residues and formylated N-terminus methionine peptides (fMPs)^31^. Moreover, their binary fission and antibiotic sensitivity supporting the endosymbiotic theory of the shared evolutionary past between mitochondria and bacteria^32^. The idea that mitochondria may originate from bacteria has been proposed in the early 20^th^ century^33^, ^34^. In 1967, American scientist Lynn Margulis (then Lynn Sagan) published the famous article “On the Origin of Mitosing Cells”, in which mitochondria and chloroplasts are proposed to be evolved from endosymbiotic bacteria^35^. Since them, growing evidence from high-throughput gene sequencing and proteomic data support that mitochondria originated from an endosymbiotic event involving the uptake of a Gram-negative bacterium capable of respiring aerobically by a fermentative Asgard rchaeal host cell^36^-^38^. This endosymbiosis theory of mitochondria origin define the important evolutionary event of eukaryotic cells, as this organelle originated at the same time as the nuclear component of eukaryotic cell^39^. According to the fossil records, this endosymbiotic event likely took place prior to the divergence of all known eukaryotes between 1.5 and 2 billion years ago^40^. The co-evolutionary changes between mitochondria and eukaryotic cells result in the transition of the endosymbiotic bacterium into a permanent organelle.

As a result of endosymbiosis, the evolution of this cooperative relationship between mitochondria and eukaryotic cells made fundamental changes for both two. From one side, mitochondria retained a part of the genome originated from their bacterial ancestors. The propel mitochondrial functions require more than 1000 proteins encoded by nuclear genome^41^, and only 14-16% of the modern mitochondrial proteome can be traced back to the ancient bacteria^42^. In mammalian cells, mitochondria generally have a single copy of mtDNA and mitochondrial nucleoids are compacted by mitochondrial transcription factor A (TFAM) into an irregular ellipsoidal shape^43^. In general, mtDNA (16,569-base pair) codes for 13 mRNAs, 22 tRNAs and 2 rRNAs^44^-^46^, encoding 13 proteins that are components of the respiratory chain located in the inner mitochondrial membrane. From the other side, most eukaryotic cells possess a population of mitochondria, therefore multiple mtDNA copies. This intracellular mitochondrial heterogeneity lead to the intercellular mitochondrial heterogeneity, therefore cellular heterogeneity^47^. The mitochondrial heterogeneity contributes to the tissue specific energy demands, cell fate determination and tissue remodeling^48^, ^49^.

## Mechanisms of mitochondria transfer

As co-opted for energy production and cellular metabolism, mitochondria have been long considered as autonomous organelles with cytoplasmic inheritance^50^. It has been widely accepted that mitochondria and mitochondrial DNA genome can be transfer or segregated from parent to daughter cells during cell division. In 2004, a novel model of *in vitro* cell-to-cell communication by tunneling nanotubes (TNT) which facilitate organelle transport was reported by Gerdes and colleagues^51^. After this report, growing evidence continuously show that this TNT-like bridges can transfer mitochondria and other organelles between living cells^52^, ^53^. In 2015, Brridge and his colleagues utilized mouse tumor models and reported the primary evidence of mitochondria transfer *in vivo*, in which mtDNA from healthy host cells were transferred into tumor cells and partially restored the mitochondrial functions in tumor cells^54^.

**Figure 2:**
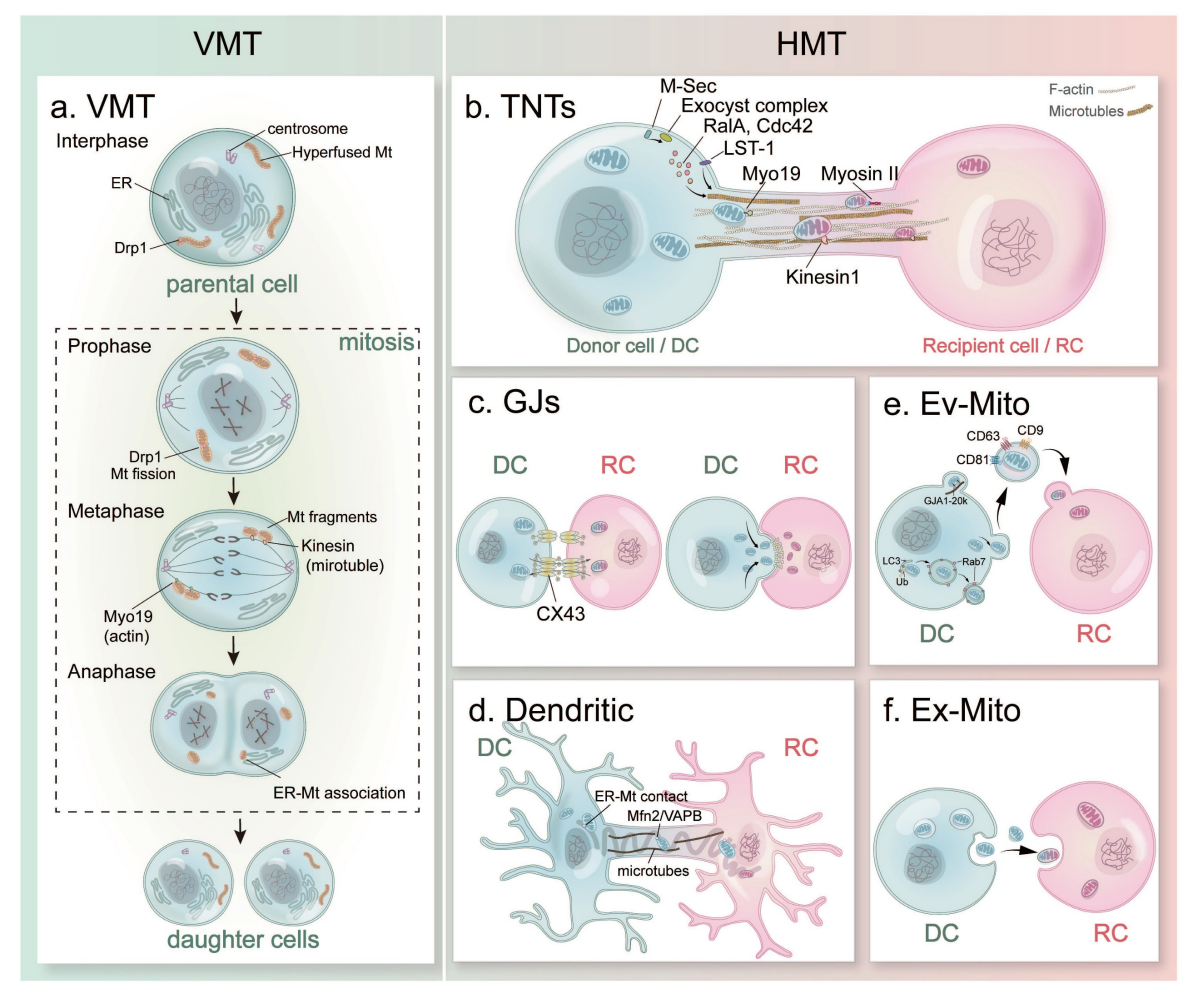
** Models of mitochondria transfer. (a)** Simplified schematic representation of the VMT mode between parental cell and daughter cells;** (b)** TNT-mediated HMT mode between donor cell and recipient cell; **(c)** GJ-mediated HMT mode; **(d)** Dendrite-mediated HMT mode; **(e)** Ev-Mito transfer; **(f)** Ex-Mito transfer. Abbreviations: VMT, vertical mitochondria transfer; HMT, horizontal mitochondria transfer; TNTs, tunneling nanotubes; GJs, gap junctions; Ev-Mito, extracellular vesicle-associated mitochondria transfer; Ex-Mito, extracellular mitochondria transfer; DC, donor cell; RC, recipient cell.

To identify these two modes of mitochondria transfer, vertical mitochondria transfer (VMT) is referring to the parent-daughter mitochondrial inheritance, and horizontal mitochondria transfer (HMT) represents the mitochondrial trafficking between living cells. Since these pioneering discoveries, increasing evidence showed that mitochondrial communications between cells occur more frequently than previously understood. Detailed mechanisms behind VMT were reported (***Figure 2. a***), and the diverse modes of HMT were discovered (***Figure 2. b-f***).

### Vertical mitochondria transfer (VMT)

During cell mitosis, VMT is critical to properly partition the functional organelles as well as nuclear acid information to daughter cell. In general, mitochondria are typically dispersed throughout the cell cytoplasm with special accumulation only during distinct cellular events, such as synaptic transmission^55^ or germ cell formation^56^. Moreover, mitochondria also undergo active intracellular movement on the actin or microtubule-based cytoskeletal network by dynein, kinesin and myosin-based motor proteins^57^-^59^. Considering the special feature of mitochondria, passing mtDNA and the functional sets of mitochondria is important to achieve successful division^15^.

Mitochondrial dynamics are achieved by a balance between mitochondrial fusion and fission: Mitochondrial fusion is the physical merging of the outer and then the inner mitochondrial membranes of two originally distinct mitochondria; whereas mitochondrial fission is the process of division of a single mitochondria into two or more independent structures. The continual fusion and fission of mitochondria is extremely critical to maintain mitochondrial integrity and homeostasis, which were systematically summarized and reviewed in published paper^60^-^64^. Here, we focused on the mitochondrial segregation during VMT process in mitosis.

Mitosis ensures genome integrity by mediating precise segregation of the duplicated genetic material, including mtDNA (***Figure 2. a***). During the interphase (G1, S, G2 phases), mitochondria form a extensively fused and interconnected tubule structure^65^. Mitochondrial fission from mitochondrial fragment in early mitotic phase by the phosphorylation of Drp1 through both Cdk1-cyclin B^66^ and Auora A mediation^67^. When cells enter the prophase, mitochondria start to prepare for their division by recruiting the pro-fission dynamin-related GTPase Drp1 and its receptors (including Mff, MiD49 and MiD51) localized on the outer membrane of mitochondria^68^. Mitochondrial highly fragmented morphology which is even more pronounced when cells reach the metaphase and anaphase stages. During the whole cell division processes, mitochondria interact with microtubules and actin cytoskeleton, as well as endoplasmic reticulum (ER) to achieve propel partitioning. On one hand, mitochondria associate with the growing microtubule tips through interactions between mitochondrial protein Miro and the cytoskeletal-associated protein Cenp-F^69^. On the other hand, Myo19 actin-based motor helps mitochondria movement and contribute to faithful segregation during division^70^. In addition, mitochondria form a membrane contact sites with ER in cells, which expand and display a tighter physical association between these two organelles during division^71^. This ER-MT association could support mitochondrial functions during mitosis and co-segregation of the two organelles to the daughter cells. At the end of cytokinesis during the telophase, mitochondria begin to recruit the pro-fusion proteins MNF1/2 at the outer membrane and OPA1 at the inner membrane and achieve elongated structures^15^, ^65^. By the time of cell division completion, the fragmented mitochondria re-form the filamentous network structure in the daughter cells after division^72^. In short summary, mitochondria remodel through complex structural changes and dynamic associations with the cytoskeleton during VMT process.

### Horizontal mitochondria transfer (HMT)

Compared to VMT, an emerging body of literature indicates that a wide range of cell types export their mitochondria for delivery to developmentally unrelated cell types through HMT approach. Even though the detailed molecular and signaling mechanisms underlying HMT are not fully understood, it has been reported to occur between adjacent cells of the same type, between distant cells of different types, and even across remote, distinct tissues. In general, HMT can be classified into two main categories: contact-dependent and contact-independent transfer.

#### Contact-dependent mitochondria transfer

The contact-dependent HMT mode refers to the formation of transient cellular connections between donor and receptor cells, through which mitochondria can move from one cell to another. Currently reported contact-dependent mitochondria transfer mechanisms include tunneling nanotubes (TNTs), gap junction (GJ)-medicated transfer and dendritic structure-mediated transfer (***Figure 2. b-d***).

##### Tunneling nanotubes (TNTs)-mediated transfer

TNTs were first reported and referred as “highways” for intercellular organelle transport in 2004 between the same type of cells, including rat pheochromocytoma PC12 cells^51^ and immune cells^73^. Later on, TNT-mediated mitochondria transfer was reported between two cells from different types and species, transferring from human mesenchymal stem cells (hMSCs) to co-cultured rat cardiac myocytes^74^. From *in vitro* to *in vivo* systems, TNTs are now extensive studied with more advanced and diverse detecting approaches, such as tissue-specific labeling method, scanning electron microscopy (SEM), atomic force microscopy (AFM), cryo-electron microscopy, *in vivo* live imaging, and others.

Mitochondria have been shown as the most frequently reported organelle that are unidirectionally or bidirectionally transferred by TNTs^75^. The components of cytoskeleton and the size (including length and diameter) of TNTs vary depending on the cell type, micro-environment and cellular status^76^. The diameter of TNTs can vary between 50 to 1500 nm, and their length can range from several tens to hundreds of micro^76^, ^77^. Reported data suggest that various stress factors, for example p53 activation^78^, that induce mitochondrial damage could facilitate TNT formation and associated mitochondria transfer.

The formation of TNTs involves a variety of molecules (***Figure 2. b***)^79^, including M-Sec, small GTPases, exocyst complex, and leukocyte specific transcript 1 (LST1). M-Sec, a mammalian protein as known as TNF alpha induced protein 2, was reported to induce de novo formation of plasma membrane protrusions through Ral-exocyst pathway during TNT formation^80^. The exocyst complex is an octameric protein complex, which has been reported with functions during the fusion of secretory vesicles derived from Golgi body with plasma membrane in exocytosis^81^. During TNT formation, M-Sec interacts with exocyst complex and promotes its assembly, during which small GTPase RalA and Cdc42 are recruited and lead to the remodeling of actin cytoskeleton^80^. LST1, a transmembrane protein, recruits RalA and the actin cross-linked protein filamin to the submembrane region, which accelerates the interaction between RalA and the exocyst complex, ultimately leading to TNT formation^82^. Cdc42 is reported with functions during the prolongation stage of TNTs^80^. Compared to the mechanism behind TNT formation, the molecular regulation of the membrane fusion between the donor and the target cells remains unclear.

Structurally, TNTs are thin membrane tubes that are filled with cytoskelston filaments, mostly F-actin and microtubles^76^. F-actin is the most common cytoskeleton found in TNTs, whose labeling is widely used to mark TNTs in cells. Besides F-actin, microtubules also participate in TNT functions. For example, cultured hippocampal neurons generated protrusions towards astrocytes and formed microtubule-containing TNTs, in which 35% of TNTs were F-actin negative under fluorescence microscopy^83^. Moreover, cells may from different types of TNTs with diverse functions. For example, human monocyte-derived macrophages can form two types of TNTs, one is thin nanotubes containing only F-actin, the other is thick nanotubes (diameter larger than 0.7 μm) containing both F-actin and microtubules^84^. Interestingly, in these cells, bacteria could be trapped and only surf along thin nanotubes after be phagocytosed; mitochondria and intracellular vesicles were detected and transferred only in thick nanotubes^84^.

Since the main cytoskeletons in TNTs include F-actin and microtubes, the shuttling of mitochondria requires these cytoskeletons associated motors for intercellular transportation. For example, F-actin polymerization and F-actin-associated myosin II motors are essential for mitochondria transfer in TNTs^85^. Selectively blocking F-actin polymerization by cytochalasin B, or treatment of myosin II specific inhibitor S-(-)-Blebbistatin inhibit TNT formation and related intercellular organelle transfer^85^, ^86^. Moreover, Miro1, a calcium-sensitive Rho-GTPase located in the outer mitochondrial membrane, is essential for F-actin and microtubule-mediated mitochondria transfer. Miro1 has been reported to interact with Myosin XIX (Myo19), an actin-based motor, which contributes to mitochondria cristae structure and potentially mitochondria transfer^58^, ^87^. Miro1 also can interact with mitofusion1/2 and combines with the kinesin-1 molecular motor through TRAK1/2 and OIP106/98, forming a mitochondria-loaded vesicle and enabling the mitochondrial transfer on microtubules in neurons^77^, ^88^, ^89^. Miro1 has been reported with essential functions in mitochondria transfer from mesenchymal stem cells (MSCs) to epithelial cells during epithelial injury and recovery^90^. *In vivo* experimental mouse study further suggests that both Miro1 and Miro2 are required for TRAK2-mediated mitochondrial motility and positioning on actin and microtubule cytoskeleton^91^.

The transport of mitochodnria transfer via TNTs is bi-directional. Interestingly, functional mitochondria in healthy cells are preferably released and transferred to damaged or injured cells. For example in brain environment, TNTs were observed between neurons, between glia cells, and between neurons and glia cells. In TNTs formed between astrocytes, an extracellular molecule S100A4 seems control TNT direction. In astrocytes, p53 activates caspase-3, which leads to the cleavage and decreased concentration of S100A4 in cells^92^. As a result, a gradient of S100A4 was formed in astrocyte population, and TNTs are initiated in low concentration cells towards high concentration cells^92^. Moreover, another team reported that when neurons were subjected to oxygen-glucose deprivation/reoxygenation exposure damage, TNT formation and mitochondrial transportation from astrocytes to injured neurons were facilitated^93^. In addition, TNTs between neurons and microglia are bi-directional, and microglia preferred transfer mitochondria to α-Synuclein burdened neurons over the healthy ones^94^.

Till now, TNT-mediated mitochondria transfer is the most common transfer route of HMT, which has been reported during physiology and pathological conditions in the immune system, cardiovascular system, respiratory system, epithelial tissues, central nervous system (CNS), and cancers^95^. Detailed mechanisms behind TNT-mediated HMTs may vary with reflection of their distinct tissue backgrounds.

##### Gap junction-mediated transfer

Gap junctions (GJs) are specialized intercellular channels between adjacent cells, allowing direct cell-cell transfer of cytoplasmic contents^96^. GJs are encoded by connexin gene family in mammalian, which has been extensively studied in the past century. Among all the connexin members, connexin 43 (Cx43) is widely expressed in mammalian tissues. Like most trans-membrane proteins, Cx43 protein is synthesized by ribosomes, bounded to the ER membrane, trafficked via the ER-Golgi mechanism, and finally inserted into the plasma membrane (PM)^97^. Once delivered to the PM, Cx43 proteins are assembled into hexamers and function as hemichannels in one cell^97^. When two cells connect, opposed Cx43 connnexins from two cells form a complete gap junction facilitating direct intercellular communication. Reported studies showed that Cx43-mediated GJs are gated open more often than closed, which opposites to Cx43-mediated hemichannels^98^.

Several reports illustrate the importance of Cx43-mediated GJs in the mitochondria transfer process^99^. In bone marrow environment, Cx43 in hematopoietic stem and progenitor cells (HSPC) mediates the mitochondria transfer from HSPCs to MSCs through cell-contact dependent approach^100^. Moreover, MSCs deliver mitochondria to damaged chondrocytes under chemical, environmental, and mechanical stress through Cx43-associated GJs in OA cartilage^101^, ^102^. *In vitro* co-culture and *in vivo* mitochondria labeling method indicated that bone marrow stromal cells (BMSCs) transfer mitochondria to injured motor neurons, which is blocked by GJ inhibitor 18β glycyrrhetinic acid^103^. In addition, ultra-purified BMSCs transfer a greater number of mitochondria into mitochondria-deficient (ρ0) cells mainly through Cx43-GJs^104^.

These Cx43-mediated mitochondria transfer may achieved by different mechanisms (***Figure 2. c***). First, Cx43 can facilitate mitochondria transfer through direct GJ-mediated cell-contact^95^. The Cx43-mediated GJ channels are non-selective pores between cells, allowing the exchanges of many intracellular molecules (up to ~1kDa), such as inorganic salts, sugars, amino acids, and nucleotides pass through connecting cells^97^, ^98^. Giving the size of Cx43-mediated GJs within a range of 10-16 Å/mesh^105^ (~1190-2000 μm) and typical mitochondria size with 0.5-3 μm^106^, it is possible that mitochondria can directly pass through these channels. Second, Cx43 may function as a stabilizer to adhere mitochondria to the docked membrane structure of donor and receptor cells^97^, ^107^, which further facilitate mitochondria transfer between cells. Third, a truncated Cx43 isoforms, GJA1-20k has been implicated in mitochondrial motility by mobilizing mitochondria along microtubules during mitochondria transfer from human mesenchymal stromal cells to stressed chondrocytes^102^.

In addition to GJ-mediated mitochondria transfer, Cx43 also contribute to TNTs and extracellular vesicles (EV)-mediated transfer. Studies showed that human induced pluripotent stem cell (iPSC)-derived MSCs form TNTs and transfer mitochondria to epithelial cells, which is dependent on the expression level of Cx43 in iPSC-MSCs and attenuates the mitochondrial dysfunctions of epithelial cells^108^. In lipopolysaccharide (LPS)-induced acute lung injury (ALI) model, BMSCs formed Cx43-containing GJs with the alveolar epithelia and released the mitochondria-containing EVs, which have been engulfed by the epithelia, thus restituting alveolar bioenergetics^107^. Interestingly, Cx43 is found present inside of the mitochondria as mitocondrial connexin 43 (mtCx43) in many pathological conditions, particularly in the heart^109^. mtCx43 is reported at the inner membrane of mitochondria in cardiomyocytes and contributes to mitochondrial K(+) flux, potentially by forming hemichannel-like structures^110^. Furthermore, mtCx43 participates in shRNA-Rictor-induced mitochondrial dysfunctions in the mouse embryonic stem cell-derived cardiomyocytes (ESC-CMs)^111^.

In short summary, GJ-mediated mitochondria transfer facilitates a direct and fast intercellular communications between adjacent cells. The mechanism behind Cx43-mediated mitochondrial transfer and mtCx43 functions shall be further studied in various physical and pathological conditions^99^.

##### Dendritic connection-mediated transfer

In addition to TNTs and GJs, dendritic connection has been reported to involved in mitochondria transfer in a special set of cell, osteocytes, in bone (***Figure 2. d***). Osteocytes are the terminal differentiated bone cells embedded in the mineralized bone matrix, which are inter-connected with each other with dense dendritic networks^112^. The length, density and dynamics of osteocyte dendrites are tightly associated with osteocyte functions during bone remodeling and mechanotransduction^112^-^115^. These are two main types of cytoskeleton composed in osteocyte dendrites, i.e., F-actin filaments and microtubules. Recently, Gao and his research team observed the distribution of mitochondria along the tubulin track of dendrites in the primary osteocytes, whose number decreased with aging^116^. Under live imaging microscopy, they further reported that the dynamic mitochondria transfer takes place between osteocytes through their dendrite connections, and the transferred mitochondria are able to restore the cellular metabolism in the stressed osteocytes^116^. This process requires tethering proteins, including mitofusion 2 (Mfn2) and vesicle-associated membrane protein B (VAPB), which facilitates ER-mitochondria contact formation and mediate the transfer of mitochondria between osteocytes^116^. Published reported revealed that ER can slide on stable acetylated microtubles, and the ER-mitochodria contact maintained during dynamic morphological processes in cell^117^. These results may suggest that the mitochondria transfer is achieved by the interaction between ER, mitochondria and microtubules through the cytoskeleton extension during osteocyte dendritic formation process^77^.

Even though F-actin and microtube are largely involved in HMT transfer, large numbers of HMT reports based on these cytoskeleton structures were through TNT-mediated mode, but mere study reported the cell dendrite-mediated mitochondria transfer. Till now, mitochondria transfer between osteocytes^116^, transfer from osteocytes to endothelial cells^118^, and from astrocytes to neurons^119^, are reported through cell dendrites. Structually, it is difficult to distinguish TNT-mediate and dendrite-mediate HMT mode^120^. More evidence is needed to demonstrate this dendrite-mediated transfer approach may server as a more universal mode for mitochondria communications between other types of cells with dense dendries. In addition, it is of importance for future studies is to distinguish the fundamental regulatory and transnational differences between dendritic connections and other forms of mitochondria transfer.

##### Contact-independent mitochondria transfer

Besides the cell-contact dependent mechanisms listed above, mitochondria and their DNA components can be released outside the cell with in intact, fragmented, and vesicle encapsulated forms under both physiological and pathological conditions^1^, ^121^-^123^. These contact-independent mitochondria transfer can be further categorized as two modes: i.e., extracellular vesicles with mitochondria (Ev-Mito) and extracellular mitochondria (Ex-Mito) (***Figure 2. e, f***).

##### EV-associated mitochondria (Ev-Mito) transfer

Intercelluar mitochondria transfer can be achieved by the release and capture of extracellular vesicles (EVs), i.e., Ev-Mito mode, as the second common mechanism reported beside TNTs (***Figure 2. e***).

Based on their size, EVs can be further divided in to three subtypes: exsosomes (30 - 100 nm), microvesicles (MVs, 100 nm - 1 μm), and apoptotic bodies (˃ 1 μm)^77^, ^124^. As limited in size, exsosomes derived from endosomal cell membranes only contain mtDNA and other small molecules^125^. Small EVs (100-200 nm), marked with the tetraspanins CD63, CD9, and CD81, also contains damaged mitochondrial components^95^, ^126^, ^127^. Large MVs (~ 1 μm size) can load functional mitochondria, which may formed via multivesicular bodies (MVBs) or bud off from the plasma membrane^95^. This Ev-Mito transfer mode has been reported in various types of cells under both physical and pathological condition. Brown adipocytes remove damaged mitochondrial components through Ev-Mito process^127^. In bone, osteoblasts release Ev-Mito to osteoprogenitor cells and promote bone formation^128^. In the brain following ischemic stroke, astrocytes release Ev-Mito to the hypoxic neurons to support neuronal survival^119^. Moreover, cardiomyocytes also release 200-600 nm mitochondria in EVs^129^.

Even though EVs from Ev-Mito transfer are all marked with the tetraspanins CD63, CD9, and CD81, but their export from donor cells may through various export mechanisms^95^. Considering the size of extracellular mitochondria, it is originally suggested that Ev-Mito was released as microveriscles directly through plasma membrane blebbing^130^, ^131^. Moreover, live cell imaging also indicates that Ev-Mito transfer can be achieved through an exosomal/endolysosomal mechanism, in which Ev-Mito released from MSCs is incorportated into LC3-positive autophagosomes before being released^132^. Recent studies also suggest that the formation and transportation of Ev-Mito is associated with mitophagy process, the key regulation for mitochondrial quality control by promoting lysosomal degradation of damaged mitochondria, through both PINK1/Parkin-dependent and -independent pathway^133^. Data showed that the activation of Parkin targets damaged mitochondrial content to lysosomes for degradation thus preventing their inclusion into EVs^134^. Moreover, thermogenically stressed brown adipocytes release Ev-Mito that contain oxidatively damaged mitochondrial proteins through PINK1-dependent manner and exportation requires PARKIN protein^127^, ^135^. Moreover, overexpression of Parkin-independent mitophagy regulator, BNIP3 (BCL2 interacting protein 3) and BNIP3L/NIX (BCL2 interacting protein 3 like) prevents the release of Ev-Mito^136^. In addition, another study reported that small GTPase Rab7 is essential for the secretion of mitochondria-contained multivesicular bodies (MVB) in the heart of aged mouse during cardiac pathophysiology^129^.

Ev-Mito transfer has emerging as the second common HMT mode reported in various tissues and organs. Since the EV-mediated communications are not limited to two adjacent cells, their influence can be reach out to broad internal environment and achieve fundamental homeostasis.

##### Extracellular mitochondria (Ex-Mito) transfer

Free or naked mitochondria can be transferred between donor and recipient cells, which is called extracellular mitochondria (Ex-Mito) transfer (***Figure 2. f***). Since mitochondria, as independent organelle, have its own double membranes, cell-free mitochondria can be released or extruded into the circulating system as Ex-Mito^95^. Reported data showed that free Ex-Mito have a size approximately 0.5-1 μm in diameter, which lack EVs and contain a full-length of mtDNA genome^121^, ^137^. The free mitochondria were first identified in the blood from both mice and humans, in which the activated platelets contain both free Ex-Mito and Ev-Mito in a ~2:1 ratio^138^.

Mechanistically, the extrusion and internalization of free mitochondria mostly occur during the process for mitochondrial quality control or when cells are under stress^77^. For example, cytoplasmic vacuoles engulf mitochondria and subsequently fuse with the plasma membrane, leading to Ex-Mito release to the extracellular medium during tumor necrosis factor α (TNFα)-induced cell death in a caspase-dependent fashion^139^. Moreover, Hela cells extrude fragments of mitochondria under high rates of ROS conditions^140^. *In vivo*, plates extrude Ex-Mito to enhance inflammatory responses^138^. Moreover, adipocytes also generate Ex-Mito locally and transferred to macrophages or into the circulation for distant organs^137^. With growing evidence to link free mitochondria or mitochondrial contents with pathological conditions, the study of Ex-Mito could provide a new way to find mitochondrial associated biomarkers for diseases.

## Mitochondria transfer in tissue homeostasis

Communication between cells is crucial for maintaining life activities and coordinating the normal operation of various organs and systems. Mitochondria transfer is an important form of intercellular communication, which supports the metabolism of recipient cells, controls the mitochondrial mass of donor cells, regulates the immune system, and maintains metabolic homeostasis in various tissue/organ backgrounds^123^, ^141^. Here, we summarize current progresses of mitochondria transfer in different systems (***Table 1***) and highlight their essential regulatory mechanisms during physiological conditions and pathogenesis.

### Immune systems

Inflammation has been associated with various degenerative and acute diseases, as well as the aging process, with mitochondrial alterations playing a central role in these phenomena^142^. Beyond their bioenergetic and biosynthetic functions, mitochondria are increasingly acknowledged as a key trigger of innate immunity that influence antimicrobial responses, autoimmune diseases, metabolic disorders, and cancers^143^. When mitochondria are compromised due to damage or mutations, excess ROS are produced, and cellular stress cannot be effectively resolved. Consequently, mitochondria serve as principal regulators of the pro-inflammatory states, modulating the innate immunity through redox-sensitive inflammatory pathways or by directly activating the inflammasome^144^.

Reported studies showed that mitochondria transfer is involved in immune responses. For example, platelet-derived mitochondria that transfer to human dermal fibroblasts elicit an anti-inflammatory process, potentially by suppressing both intracellular and mitochondrial ROS production and facilitating the wound repair^145^. In bone marrow, stromal cell-derived mitochondria are delivered to progenitor cells, inducing a leukocyte proliferative response that defends against acute bacterial infections at distant sites^146^. MSC-mediated mitochondrial transfer through EVs, which can alter alveolar macrophage phenotype, enhancing their anti-inflammatory and phagocytic functions, while promoting tissue regeneration^147^, ^148^.

Moreover, mitochondria transfer also participates in pathological immune-responses. Data showed that extracellular mtDNA can promote NLRP3 inflammasome activation and induce acute lung injury through TLR9 and NF-κB^149^. In autoimmune diseases, mitochondria transfer from MSCs is associated with enhanced regulatory T cell activation, leading to increased expression of FOXP3, IL2RA, CTLA4, and TGF-β1, thus contributing to the suppression of immune responses and the enhance of immune tolerance^150^. Macrophages, typically responsible for digesting dysfunctional mitochondria to maintain homeostasis, can also donate mitochondria in pathological contexts. In osteoporosis, macrophages with an M1-like phenotype transfer mitochondria to MSCs, inducing ROS bursts that impair the osteogenic differentiation of MSCs^151^. Conversely, M2-like macrophages facilitate the resolution of inflammatory pain via transferring mitochondria to sensory neurons, mediated by the CD200 receptor (CD200R) on macrophages and its noncanonical ligand iSEC on sensory neurons^152^. Natural killer T cells transfer mitochondria via TNTs, enhancing cancer cell basal respiration, spare respiratory capacity, and growth^153^.

### Nervous system

The central nervous system (CNS) comprises the brain and spinal cord. At the cellular level, CNS contains neurons, glial cells, and other cell types^154^. Mitochondria are integral to the function of the CNS as they produce the majority of the energy required for membrane ATPases, the influx and efflux of neurotransmitters, and the formation of new neural circuits^155^. Mitochondria exhibit an adaptive response to the fluctuating demands of their host cells to maintain bioenergetic and oxidative homeostasis^156^. In the context of a damaged or diseased brain, mitochondrial dysfunction results in decreased ATP levels^157^, which further triggers an increase in ROS production and the activation of related apoptosis pathways^158^. Therefore, various forms of mitochondrial dysfunction have been identified in conditions such as schizophrenia (SZ)^159^, spinal cord injury^160^, Parkinson's disease^161^, and Alzheimer's disease^162^.

To achieve the homeostasis in nervous systems, mitochondria transfer has been reported between different types of cells. Data showed that multipotential mesenchymal stem cells (MMSCs) can transfer mitochondria to both neuron and glial cells^163^. Moreover, astrocytes can transfer mitochondria to neurons, which further modify the calcium concentration in damaged neurons^164^. In response to ischemic injury, glial cells can transfer healthy mitochondria into injured neurons, thereby enhancing the survival rate of the damaged cells^165^. Furthermore, mitochondria can be transferred from astrocytes to microglia, influencing their phagocytic functions; conversely, microglia can also transfer mitochondria to astrocytes, which may either promote or inhibit neuronal inflammation^94^.

Currently, the restoration of normal biological function in damaged mitochondria is one of the research hotspots for treating neurological diseases^166^. Numerous studies have confirmed that mitochondria transfer can alter the biological activity and physiological state of recipient cells in spinal cord injury (SCI) models^167^. The transfer of normal mitochondria from donor cells to recipient cells with abnormal mitochondrial functions can enhance mitochondria-related biosynthesis in recipient cells, and affect their biological functions^168^. Studies have demonstrated that transplanted BMSCs can prevent neuronal apoptosis and facilitate locomotor functional recovery by transferring mitochondria to injured neurons following SCI^103^. Furthermore, result indicate that photobiomodulation (PBM) intervention facilitate the transfer of platelet-derived mitochondria to neurons in the treatment of SCI, which is more effective than single treatment in terms of motor function recovery, tissue repair, and inhibition of neuronal apoptosis^169^. Therefore, mitochondrial transplantation represents a promising therapeutic approach for spinal cord injury. In context of neurological ischemic diseases or neurodegenerative diseases caused by ischemia and hypoxia, the transfer of mitochondria from healthy cells to damaged cells can often restore cellular activity, rescue mitochondrial respiratory function, promote oxidative phosphorylation, reduce lactic acid production, and ultimately prevent apoptosis^170^. Regarding SZ, isolated active normal mitochondria can enter various cell types without any manipulations, thereby improving impaired mitochondrial functions in SZ-derived cells^171^. In neurological disease, cerebrospinal fluid (CSF) lactate levels are elevated in stroke patients and show a negative correlation with astrocytic mitochondria. Inhibition of mitochondria transfer from astrocytes to injured neurons exacerbates ischemia-reperfusion injury in mouse models of ischemic stroke, whereas promoting the transfer of astrocytic mitochondria to neurons can counteract ischemic stroke^119^, ^172^, ^173^.

### Cardiovascular system

Mitochondria make up to approximately 29% to 36% of cardiac myocyte volume, thus providing energy for the contraction of myocardial cells and critical for cardiac functions^174^. Therefore, maintaining the structural integrity and functional stability of mitochondria is of crucial significance for the overall health of the cardiovascular system^175^. The balance of mitochondrial homeostasis is regulated by dynamic processes, such as mitochondrial dynamics, crest remodeling, biosynthesis, autophagy, and oxidative stress. When mitochondrial homeostasis is disrupted, the contractile function of myocardial cells is affected and their activities are hindered, which can lead to diseases, such as cardiomyopathy, atherosclerosis, hypertension and heart failure^176^.

Mitochondria transfer is a key example of intercellular communication in cardiomyocytes^177^, which supports the metabolism of recipient cells and regulate tissue homeostasis^120^. The methods for treating cardiovascular diseases through mitochondrial transplantation include naked mitochondria transfer and cell-mediated mitochondrial transplantation, the latter of which is mainly mediated by TNTs, EVs and cell fusion^178^. In the heart, following myocardial ischemia or reperfusion injury, cardiomyocyte-derived small EVs deliver mitochondrial components to cardiac fibroblasts, activating the cGAS-STING pathway and promoting ischemic cardiac fibrosis^179^. Additionally, after myocardial infarction, cardiac fibroblasts transfer damaged mitochondria via small EVs to macrophages, activating the NLRP3 pathway and exacerbating tissue inflammation and adverse ventricular remodeling^180^.

Moreover, the potential of mitochondria transfer therapy in restoring the cellular function of myocardial cells has been tested as an important approach in the treatment of heart failure. For example, Zhang A injected human colon carcinoma cell lines into the hearts of wild-type mice to ensure that human mitochondria were absorbed by mouse cardiomyocytes. These results indicated that mitochondria transfer had a protective effect on doxorubicin-induced heart failure and could protect cardiomyocytes from doxorubicin-induced mitochondrial dysfunction^181^. Jin N transferred mitochondria from human umbilical cord mesenchymal stem cells (ucMSCs) into cardiomyocytes, which protects the cardiac function and prevents cardiomyocyte apoptosis, providing a new strategy for the treatment of heart failure^182^. Moreover, Sun X constructed myocardial cell injury models *in vitro* and implemented different mitochondrial transplants to explore new therapeutic measures for improving cardiovascular diseases. Studies have found that Alda-1 stimulation significantly enhances the respiratory and mechanical functions of myocardial cells caused by mitochondria transfer, showing great potential in the treatment of myocardial ischemia-reperfusion injury^183^. In addition, Lin RZ reported that mitochondria transfer between mesenchymal and endothelial cells through TNTs had a protective effect on the function of endothelial cells by triggering mitochondrial autophagy^184^.

### Respiratory system

Mitochondria transfer provides a new therapy for treating respiratory diseases caused by mitochondrial dysfunctions, such as chronic obstructive pulmonary disease and pulmonary fibrosis^185^. *In vitro*, Sinclair KA co-cultured epithelial cells with MSCs, and found that MSCs from lung tissue can transfer mitochondria to bronchial epithelial cells through multiple mechanisms, contributing to the repair of damaged bronchial epithelial cells^186^.* In vivo*, reported experimental animal studies demonstrate that mitochondria transfer serves as functional treatments in various respiratory diseases.

Asthma is a chronic respiratory disease, in which exosomes may mediate important immunomodulatory roles in asthma. To explore the potential mechanism of immune regulation by airway myeloid-derived regulatory cells (MDRC) in asthma, Hough KP transferred mitochondria to T cells through exosomes. Their data indicated that the mitochondria transfer between airway myeloid-derived regulatory cells represented a new immunomodulatory pattern in asthma, and also confirmed that exosomes were likely involved in the immune response of MDRCs in asthma^187^. This study provides a theoretical basis for the development of treatments for asthma targeting inflammatory responses. Moreover, chronic obstructive pulmonary disease (COPD), as the eighth leading cause affecting global health, is a common chronic disease characterized by airflow obstruction. Reported studies show that there are variations in the mtDNA genome in the lung tissue and peripheral blood of patients with COPD, and these variations may lead to abnormal mitochondrial functions^188^. Frankenberg GJ transferred mitochondria from MSCs to airway smooth muscle cells and found that they could regulate bioenergetics and cellular functions^189^. This also proves that the uptake of MSCs-derived mitochondria by airway smooth muscle cells can reverse mitochondrial dysfunction induced by oxidative stress, providing a theoretical basis that mitochondrial transfer can be used as a research method to explore mitochondrial dysfunction in COPD. Furthermore, pulmonary fibrosis (PF) is a serious lung disease characterized by fibroblast proliferation and a large amount of extracellular matrix deposition. Previous studies have shown that mitochondrial dysfunction in lung cells is one of the main causes promoting PF^190^. The use of exogenous mitochondria to supplement damaged mitochondria has been proposed as a strategy for treating PF. Huang T treated human placenta-derived MSCs with a combination of pioglitazone (Pg) and iron oxide nanoparticles, which could effectively transfer mitochondria to damaged lung cells and restore mitochondrial homeostasis^191^. In addition, Zhang F found that the regulation of TFAM expression in MSCs plays a key role in improving the permeability barrier of pulmonary microvascular endothelial cells (PMVECs) by mediating mitochondria transfer through TNTs, providing a new therapeutic strategy for the treatment of acute lung injury caused by sepsis^192^.

### Liver

The liver is an important metabolic organ responsible for detoxification, bile production, nutrient storage, and regulation of diverse metabolic processes. Mitochondrial quality control (MQC) plays a crucial role in maintaining liver health and function, and its disruption has been implicated in a wide range of liver diseases^193^. For example, impaired mitochondrial function is associated with hepatic lipid accumulation, oxidative stress, insulin resistance, and inflammation^194^. These pathological factors contribute to the development and progression of liver diseases such as nonalcoholic fatty liver disease (NASH), alcohol-associated liver disease (ALD), drug-induced liver injury (DILI), and viral hepatitis. Studies have shown that the AMPK pathway plays a key role in regulating hepatic energy metabolism and cellular viability^195^, and transferring healthy mitochondria can improve the symptoms of metabolic syndrome, including hypertension, hyperlipidemia, and fatty liver^196^.

Mitochondria-targeted interventions have emerged as promising therapeutic strategies for NASH, focusing on improving energy metabolism, antioxidant effects, and mitochondrial quality control. MSCs transfer mitochondria to liver cells through TNTs, which can further reduce the lipid load of the liver, promote the transformation of lipids from storage to utilization, and improve the ability of tissue homeostasis^196^, ^197^. In arsenic-induced liver injury, functional mitochondria were transferred between cells through TNTs, achieving mutual supplementation of mtDNA between cells. This compensated for the mitochondrial dysfunction caused by arsenic and hepatocyte senescence and liver damage^198^. Umbilical cord-derived MSCs (UC-MSCs) have been found to alleviate liver damage by restoring mitochondrial functions through activation of the Nrf2/HO-1 pathway^199^. In hepatic ischemia-reperfusion injury, MSC-derived EVs also deliver functional mitochondria to neutrophils, repair their mitochondrial function, and inhibit local neutrophil extracellular traps (NETs) formation, exhibiting significant nanotherapeutic effects^200^. Additionally, HMT occurs between hepatocellular carcinoma (HCC) cells via TNTs; the transfer of mitochondria from highly invasive to less invasive cells can increase the migratory and invasive capacity of recipient cells, with hypoxia further promoting this process^201^.

### Kidney

The kidney is one of the most energy-consuming organs in the human body, second only to the heart in mitochondrial content and oxygen consumption^202^, ^203^. Due to the exceptionally high oxidative activity of renal mitochondria, the kidney is highly susceptible to oxidative stress damage, thereby promoting the onset and progression of kidney diseases and potentially resulting in renal failure. Mitochondrial dysfunction inevitably leads to energy deficiency in renal tubular epithelial cells, consequently causing renal dysfunction^204^. Mitochondrial dysfunction in kidney cells can lead to various pathological conditions, including acute kidney injury (AKI) and chronic kidney disease (CKD), by affecting cellular energetics, redox balance, and cell death pathways^203^, ^205^. Therefore, maintaining mitochondrial homeostasis and quality control is essential for preserving normal renal functions.

Mitochondria play a central role in the pathogenesis and progression of kidney diseases primarily through several mechanisms, including the regulation of ROS production, immune responses, and energy metabolism^206^, ^207^. Recent studies have revealed that EVs possess multiple biological effects in kidney diseases, such as anti-inflammatory, anti-apoptotic, pro-angiogenic, and anti-fibrotic actions^208^, ^209^. Furthermore, Naoto K showed that BM-MSCs improve impaired proximal tubular epithelial cells (PTECs) in diabetic nephropathy (DN) via mitochondria transfer^210^. In this study, transferred mitochondria inhibited PTECs apoptosis, regulated related factors to inhibit ROS production, restored transporter expression, and repaired renal tubular structure. Moreover, MSCs also transfer mitochondria to macrophages, enhancing their anti-inflammatory properties and alleviating kidney injury in diabetic nephropathy through PGC-1α activation^211^.

### Musculoskeletal system

Mitochondria play a crucial role in the musculoskeletal system, influencing cellular metabolism, energy production, and homeostasis. Mitochondrial dysfunction has been implicated in various musculoskeletal conditions, including rheumatic diseases, age-related muscle loss, and osteoporosis^212^. In recent years, research on HMT within the musculoskeletal system has primarily focused on osteocytes and chondrocytes.

In osteocytes, mitochondria transfer occurs through dendritic networks, mediated by endoplasmic reticulum (ER)-mitochondrial contact and regulated by Mitofusin 2 (Mfn2)^116^. Osteocytes exchange mitochondria through interconnected dendritic networks^116^, and this feed-forward mechanism promotes bone matrix formation and restores metabolic function in stressed cells. Moreover, clinical data showed that bone is one of the most common sites of tumor metastasis. During bone metastasis, osteocytes transfer mitochondria to metastatic cancer cells and trigger cGAS/STING-mediated anti-tumor responses^116^, ^213^.

In chondrocytes, mitochondrial dysfunction is closely associated with cartilage damage. Elevated mitochondrial electron transport chain activity and increased ROS levels in injured chondrocytes result in excessive free radical and metabolite release, thereby triggering apoptosis and necrosis^214^. Due to the avascular nature of cartilage, its intrinsic ability for self-repair is limited, making restoration of chondrocyte function particularly critical for tissue repair^215^. MSC transplantation not only alleviates symptoms of osteoarthritis (OA) but also restores mitochondrial functions in damaged cells by transferring mitochondria to chondrocytes through EVs or GJs^101^, ^216^. The transfer of healthy mitochondria from MSCs to OA chondrocytes has been demonstrated to improve mitochondrial function, reduce cell apoptosis, and increase the production of cartilage-specific proteins^217^. In addition, mitochondrial transplantation can upregulate the expression of PGC-1α, downregulate Mfn2 expression, and increase mitochondrial DNA content, suggesting that mitochondria transfer promotes both mitochondrial function and biogenesis in chondrocytes^218^.

Besides osteocytes and chondrocytes, recent studies have shown that macrophages can enhance the osteogenic differentiation of MSCs through mitochondria transfer and regulation of ROS. Macrophages deliver functional mitochondria to MSCs, promoting their osteogenic differentiation and regulating bone homeostasis^219^. Moreover, osteoblasts transfer mitochondria to osteoprogenitor cells, stimulating their differentiation into mature osteoblasts^128^. In short conclusion, mitochondrial transfer in musculoskeletal system not only facilitates the osteogenic differentiation and bone formation, but also prevents osteocyte damage and suppresses chondrocyte apoptosis, highlighting its therapeutic potential in skeletal and joint diseases.

### Adipose tissue

Adipose, a connective tissue composed of mature adipocytes, fibroblasts, endothelial cells, macrophages, matrix cells, immune cells, and mesenchymal stem cells, plays a key role in regulating pathophysiological processes by influencing insulin sensitivity, blood pressure, endothelial function, fibrinolytic activity, and inflammatory responses^220^. This is dependent on both its endocrine capacity and intercellular mitochondrial communication. Under normal conditions, adipocytes can share their mitochondria with adjacent macrophages in white adipose tissue, promoting the differentiation of specific macrophage subgroups and supporting metabolic homeostasis. The ability of macrophages to take up mitochondria may be related to the expression level of heparan sulfate on their cell surface^221^. Moreover, macrophages also transfer mitochondria to adipocytes via EVs. Studies showed that M2 macrophages can transfer more mitochondria to adipocytes than M1 macrophages, promoting adipocyte-myofibroblast transition through the activation of TGF-β and PAI-1 pathways^222^. However, under obesity conditions, adipocytes release EVs containing damaged mitochondria in response to intense energy stress. These vesicles are taken up by cardiomyocytes through the circulatory system. The EV-Mito induce transient mitochondrial oxidative stress in cardiac tissue, preconditioning the heart and protecting it from ischemia or reperfusion injury^127^.

Growing evidence has shown that mitochondria from adipose-derived mesenchymal stem cells (ADSCs) are beneficial for certain diseases. For instance, ADSCs connected with macrophages through TNTs to deliver mitochondria to synovial macrophages, alleviating rheumatoid arthritis^223^. Moreover, ADSCs transferred mitochondria to dendritic cells (DCs) via EVs. After internalizing the functional mitochondria from EVs, DCs activated the MAPK/ERK1/2/FOXO1/autophagy pathway, altering cell metabolism. This reprogramming facilitated the shift of DCs from an activated state to a tolerant state, thereby reducing inflammation^224^. In an *in vitro* insulin secretion study, ADSC mitochondria were transferred to pancreatic islet cells via TNTs to improve insulin secretion function^225^. Moreover, mitochondria were transferred through TNTs into rat cardiomyocytes three days after ADSCs were transplanted onto the surface of ischemic hearts, improving the cardiac function in rat models of ischemic cardiomyopathy^226^. However, the HMTs of ADSCs is not always beneficial, but displays a harmful effect. For example, ADSCs cocultured with different breast cancer cells (BCCs) transferred mitochondria to BCCs via TNTs, increasing ATP production and driving ABC transporter-mediated multidrug resistance (MDR)^227^.

### Reproductive system

The reproductive system plays a pivotal role in species continuation and genetic transmission. Mitochondria, serving as the cellular powerhouses and subject to maternal inheritance, critically influence gamete quality and embryonic development. Age-associated mitochondrial dysfunction is a significant contributor to declining gamete quality and infertility. Recent studies demonstrates that autologous mitochondrial transplantation (MRT) - the supplementation of viable mitochondria into aged or compromised oocytes - is an effective strategy for rescuing fertility potential.

In aged mouse models, transplantation of mitochondria derived from autologous sources such as adipose-derived stem cells (ADSCs)^228^, endometrial mesenchymal stem cells (EnMSCs)^229^, or induced pluripotent stem cells (iPSCs)^230^ significantly enhanced oocyte quality, promoted embryonic development, and increased live birth rates. Human studies further evaluated mitochondria from various autologous stem cells (ADSCs, BMSCs, urine-derived stem cells (USCs), and ovarian granulosa cells (GCs)). Notably, mitochondria from USCs exhibited superior characteristics, including a characteristic non-fused spherical morphology, high abundance, low ROS levels, and a robust coupled metabolic profile reliant on both glycolysis and oxidative phosphorylation (OXPHOS). Following transplantation into human oocytes, USC mitochondria effectively restored mitochondrial content and function, ameliorated metabolic status, improved embryonic euploidy rates, and supported normal embryonic development^231^. Furthermore, MRT has proven effective in mitigating mitochondrial damage and oxidative stress induced by oocyte cryopreservation - a routine procedure in assisted reproductive technology (ART) - thereby restoring developmental competence and live birth potential of frozen-thawed oocytes^232^.

These collective findings highlight the considerable promise of autologous mitochondrial transplantation, particularly utilizing optimized sources like USCs, for: (i) improving the function of aged or damaged oocytes, (ii) treating associated infertility, and (iii) enhancing the success rates of ART procedures, including oocyte cryopreservation.

In short summary, HMT has been emerged as a promising mechanism for tissue revitalization, offering potential therapeutic strategies for treating tissue damage and degeneration. Understanding the dual nature of mitochondria transfer in health and disease provides new opportunities for targeted intervention. Mitochondria transfer has a unique advantage in the study of tissue homeostasis and in the treatment of systematic diseases, providing researchers with a new perspective to explore treatment plans for various diseases.

## Functions of mitochondria transfer

**Figure 3:**
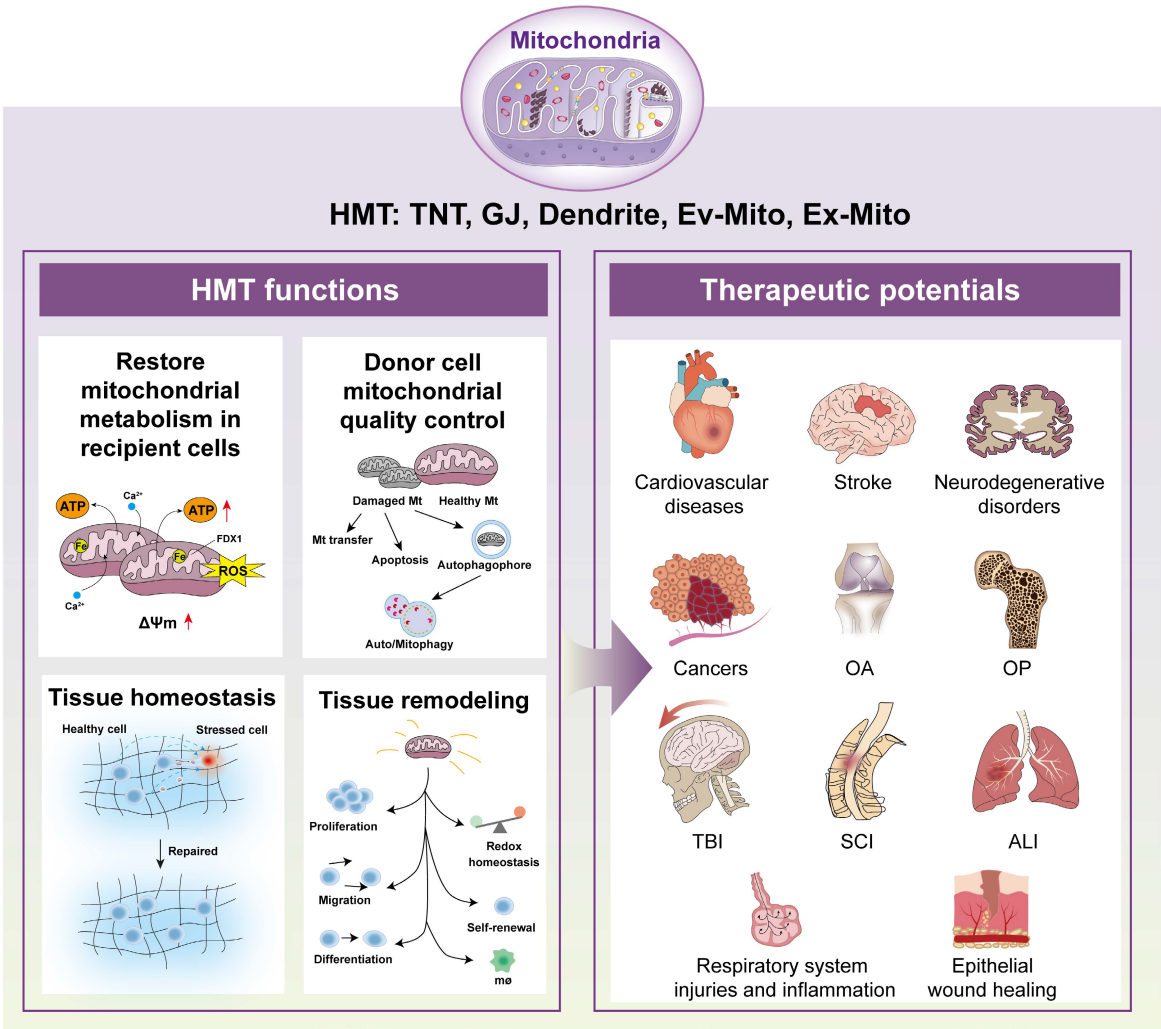
** Schematic diagram of functions and therapeutic potentials of mitochondria transfer.** Mitochondria transfer has been shown with essential functions in the pathology and treatment of various tissues and diseases. These functions include restoration mitochondrial metabolism in recipient cells, donor cell mitochondria quality control, tissue homeostasis and tissue remodeling, which have been demonstrated with essential roles in various disease pathogenesis and treatment. Abbreviations: VMT, vertical mitochondria transfer; HMT, horizontal mitochondria transfer; TNTs, tunneling nanotubes; GJs, gap junctions; Ev-Mito, extracellular vesicle-associated mitochondria transfer; Ex-Mito, extracellular mitochondria transfer; OA, osteoarthritis; OP, osteoporosis; TBI, traumatic brain injury; SCI, spinal cord injury; ALI, acute lung injury.

The dynamic intercellular exchange of mitochondria plays a crucial role in cell and tissue physiology^95^,^233^. Mitochondria transfer serves multiple functions, including restoring mitochondrial metabolism, maintaining donor cell quality control, promoting tissue homeostasis, and promoting tissue remodeling^77^. In the following sections, we explore these roles in detail and schematically summarize the mechanisms and outcomes of mitochondrial transfer on recent advances (***Figure 3***).

### Restore mitochondrial metabolism in recipient cells

HMT represents a fundamental mechanism by which donor cells—including MSCs, astrocytes, and immune cells—restore or augment mitochondrial function in stressed or injured recipient cells. This process rescues mitochondrial metabolism by replenishing the mitochondrial pool, restoring membrane potential, ATP production, and reducing oxidative stress, thereby mitigating cell death across various pathological contexts such as myocardial infarction, stroke, acute lung injury, and neurodegenerative disorders^2^, ^234^. Transferring mitochondria from neighboring cells or foreign cells can alleviate mitochondrial dysfunction and restore its biological function^235^, ^236^. Transfer of mitochondria or mitochondrial components from MSCs or their EVs to alveolar cells restores bioenergetic function, as evidenced by increased alveolar ATP levels, enhanced mtDNA content, improved mitochondrial membrane potential, and elevated OXPHOS activity. Notably, only EVs containing mitochondria confer significant protection in models of lung injury^107^, ^147^, ^237^.

Mitochondria transfer to recipient cells can restore mitochondrial metabolism by replacing damaged mitochondria with healthy ones, thereby improving energetic balance, calcium and iron homeostasis, and reducing oxidative stress—functions that are particularly beneficial in neurodegenerative disease^238^. For example, CD8⁺T cells acquiring exogenous mitochondria display enhanced mitochondrial respiration and spare respiratory capacity, leading to improved expansion, tumor infiltration, and reduced exhaustion in tumor-bearing hosts^239^. Moreover, intercellular nanotube-mediated mitochondria transfer from immune cells to cancer cells markedly enhances mitochondrial metabolism in recipient cancer cells, as reflected by increased mitochondrial respiration, basal respiration, and spare respiratory capacity^153^. Traumatic brain injury impairs mitochondrial respiration in recipient neurons, resulting in reduced oxygen consumption and compromised ATP production^240^. These findings suggest that donor cells can induce long-term behavioral changes in recipient cells or tissues by transferring relatively small numbers of mitochondria.

### Donor cell mitochondrial quality control

In addition to being involved in supporting the metabolism of recipient cells, mitochondria transfer also serves as a quality control mechanism for donor cells, allowing them to maintain their own mitochondrial health^95^. By offloading damaged or dysfunctional mitochondria into recipient cells or extracellular vesicles, donor cells can reduce oxidative stress and prevent the accumulation of defective organelles^241^. This process is tightly regulated and often involves mitophagy pathways, where damaged mitochondria are tagged for transfer or degradation^242^. This process has been observed in stem cells, neurons, immune cells, and cancer cells, and may involve mechanisms such as mitophagy, autophagy, and lysosomal degradation. For example, brown adipocytes and cardiomyocytes release damaged mitochondrial components in extracellular vesicles, which are subsequently captured and degraded by tissue-resident macrophages^243^, ^244^. Neurons can also release damaged mitochondria and transfer them to astrocytes for disposal and recycling^119^. During acute ischemic stroke, loss of blood flow impairs mitochondrial oxidative phosphorylation and causes bioenergetic stress. HMT helps maintain mitochondrial function and supports neurovascular unit homeostasis^245^. This intercellular mitochondrial quality control adds a new layer to the regulation of cellular homeostasis and may underlie certain aspects of stem cell function and tumor progression.

### Tissue homeostasis

Beyond individual cellular effects, mitochondria transfer contributes to the maintenance of tissue homeostasis. By distributing functional mitochondria among heterogeneous cell populations, tissues can buffer local metabolic fluctuations, enhance stress resistance, and facilitate coordinated responses to injury or disease. In the central nervous system, astrocyte-to-neuron mitochondria transfer has been implicated in neuroprotection and functional recovery after ischemic insult. In hematopoietic and immune tissues, HMT helps balance redox status, sustain stem cell self-renewal, and regulate immune cell activation —processes essential for preserving tissue architecture and function under both physiological and pathological conditions.

Emerging data also indicate a role for mitochondria transfer in tissue regeneration and repair, promoting survival and proliferation of resident or recruited progenitor cells. For example, under healthy conditions, adipocytes transfer mitochondria to macrophages in white adipose tissue via macrophage surface heparan sulfate, supporting energy homeostasis^221^. Disruption of this pathway—such as during obesity or high dietary long-chain fatty acid exposure—impairs mitochondrial transfer, leading to metabolic dysfunction and release of adipocyte-derived mitochondria into the circulation, where they help counteract stresses such as metabolic and ischemic stress in distant organs such as the heart^137^.

Mitochondria transfer is also involved in disease progression and immune regulation. Transfer of mitochondria harboring cancer cell-specific mtDNA mutations to tumor-infiltrating lymphocytes induces metabolic dysfunction and senescence in T cells, impairs memory formation, and compromises antitumor immunity, thereby facilitating immune evasion^246^. In addition, through the PINK1/Parkin-Mfn2 pathway, cancer cells acquire platelet mitochondria, which reprogram them toward a metastatic phenotype by modulating the GSH/GSSG ratio and ROS levels, thereby promoting metastasis of osteosarcoma^247^. Mitochondrial transfer from MSCs restores tumor cell mitochondrial function, driving chemoresistance, heterogeneity, and metastasis in several cancers^248^-^250^.

### Tissue remodeling

Mitochondria transfer is increasingly recognized as a central mechanism in tissue remodeling—a process fundamental to development, wound healing, and adaptation to physiological stress. During tissue remodeling, cells undergo metabolic reprogramming, with mitochondria transfer providing critical energy and signaling cues to support cellular proliferation, migration, and differentiation, particularly in regenerative contexts. Notably, adipocytes have been shown to extrude damaged mitochondria into the extracellular space, where they may circulate and be taken up by recipient cells, thereby shaping the local microenvironment and modulating tissue remodeling^251^.

Emerging studies have further elucidated the complexity of mitochondria transfer in various tissue contexts. For example, platelets enhance the wound-healing capacity of MSCs by transferring platelet-derived mitochondria and inducing metabolic reprogramming^252^. Moreover, in the context of bone homeostasis, mitochondria transfer from macrophages to MSCs regulates the metabolism and osteogenic differentiation of MSCs, whereas impaired mitochondria transfer in osteoporosis disrupts this process and contributes to disease pathology^253^. These findings highlight the dual role of mitochondria transfer in tissue remodeling, with implications for both regenerative and disease processes.

## Therapeutic potentials of mitochondria transfer in diseases

Mitochondria transfer has emerged as a promising therapeutic strategy in a wide spectrum of diseases, spanning from rare mitochondrial disorders to common conditions such as cancer, tissue injury, and aging^254^. Harnessing the power of healthy mitochondria to restore metabolic capacity, modulate cellular signaling, and reshape tissue microenvironment, mitochondria-related therapeutics are reshaping translational medicine. In this section, we review the main disease categories associated with mitochondria transfer, highlight its current preclinical models and clinical therapeutic applications, and discuss key organ systems and clinical settings in which mitochondria transfer is under intensive investigation (***Table 2***).

### Inherited mitochondrial diseases

Inherited mitochondrial diseases are caused by genetic mutations affecting mtDNA or nuclear-encoded mitochondrial genes, resulting in impaired oxidative phosphorylation and multisystem dysfunction^255^. Disorders such as Friedreich's ataxia (FA), Leber hereditary optic neuropathy (LHON), Mitochondrial encephalomyopathy with lactic acidosis and stroke-like episodes (MELAS), Myoclonic epilepsy with ragged red fibers (MERRF), and Leigh syndrome (LS) typically affect high-energy-demand tissues, leading to neurological, muscular, and cardiac symptoms. Traditional treatments are mostly supportive and have limited efficacy in treating the underlying mitochondrial dysfunction^256^.

Recent advances in mitochondrial transplantation and exogenous mitochondrial delivery have shown promising results in preclinical models, providing a foundation for the development of novel therapies targeting the root causes of mitochondrial diseases. Current supplementation therapies, such as coenzyme Q10, idebenone, and EPI-743, aim to enhance mitochondrial electron transport and alleviate symptoms in selected disorders. Disease-specific interventions include high-dose L-arginine for MELAS to reduce stroke-like episodes^257^, allogeneic stem cell transplantation and enzyme replacement for mitochondrial neurogastrointestinal encephalopathy^258^, and deoxynucleoside supplementation for mtDNA depletion syndromes^259^. In addition, emerging experimental approaches focus on improving mitochondrial quality control and metabolism—for example, modulation of autophagy and mitophagy using rapamycin^260^, or enhancement of the NAD^+^ pathway with nicotinamide riboside^261^. Together, these therapies aim to alleviate symptoms, improve quality of life, and address the underlying mitochondrial dysfunction.

### Non-inherited mitochondria-related diseases

Emerging evidence suggests that mitochondria transfer also plays a crucial role in acquired and degenerative diseases^262^. In conditions such as cancer, wound healing, ischemic injury, chronic inflammation, and neurodegenerative diseases, intercellular mitochondrial exchange shapes disease progression and recovery. The therapeutic impact of mitochondria transfer in the following several key pathological conditions have been extensively studied in recent years.

#### Cancers

Cancer cells often exploit mitochondria transfer to enhance metabolic plasticity, evade apoptosis, and acquire resistance to chemotherapy or radiotherapy. Tumor-associated stromal cells, including MSCs and fibroblasts, have been shown to donate mitochondria to cancer cells via TNTs or EVs, thereby restoring mitochondrial respiration and supporting rapid proliferation^263^. Importantly, inhibiting pathological mitochondria transfer—through genetic or pharmacological means—reduces tumor growth and increases therapeutic sensitivity in preclinical models. Strategies such as selective mitochondrial depletion or disruption of TNT formation with microtubule inhibitors like vincristine (VCR) effectively impair the “rescue” function of activated MSCs within the tumor microenvironment^264^. Redox regulation is also intricately involved in this process. Thioredoxin reductase 2 (TrxR2), a key mitochondrial redox enzyme frequently overexpressed in cancer cells, confers resistance to apoptosis. Selective inhibition of TrxR2 increases mitochondrial ROS and drives the redox state toward oxidation, thereby promoting cancer cell apoptosis^265^.

Beyond pharmacological interventions, cell engineering is emerging as a promising strategy in tumor therapy. Recent work by Piekarska *et al.* demonstrates that MSCs can transfer active mitochondria to allogeneic regulatory T cells in an HLA-dependent manner, augmenting their immunosuppressive function and therapeutic potential^266^. Similarly, Court *et al.* reveal that mitochondria transfer from MSCs protects both native and engineered CAR-T cells from apoptosis, enhancing their metabolic fitness and persistence, and offering a compelling approach to improve the efficacy of T cell-based immunotherapies^267^. Recently, Zhang H *et al.* introduced a statistical deconvolution method for tracing and quantifying mitochondrial trafficking between cancer and T cells at single-cell level, which largely empowered MT research in a higher resolution^268^.

Collectively, these findings underscore the complex role of mitochondria transfer in cancer biology, highlighting both its contribution to tumor progression and its potential as a target for innovative therapeutic strategies.

#### Wound healing

Mitochondria transfer is increasingly recognized as a pivotal mechanism underpinning tissue repair and regeneration. MSCs, in particular, play an essential role in this process. Through advanced cell engineering approaches, MSCs can be optimized to transfer their mitochondria to local cells at sites of injury, resulting in elevated ATP production, enhanced cellular proliferation and migration, and accelerated tissue repair. This highlights the promise of mitochondria transfer as an innovative strategy for developing next-generation cell-derived therapies in regenerative medicine^269^. Recent studies have further elucidated the multifaceted mechanisms by which MSC-derived products exert their regenerative effects. For example, exosomes isolated from human umbilical cord MSCs (HucMSC-Exo) and injected into full-thickness skin wounds in mice have been shown to reprogram neutrophil mitochondrial metabolism, induce N2 neutrophil polarization, and thereby promote angiogenesis and enhance tissue regeneration^270^. EVs derived from MSCs also serve as efficient vehicles for mitochondria transfer in pathological settings. In diabetic wound models, MSC-derived EVs have been shown to deliver functional mitochondria to neutrophils, restoring their mitochondrial function, suppressing neutrophil extracellular trap (NET) formation and endothelial cell ferroptosis, and ultimately accelerating angiogenesis and wound healing^271^. Moreover, small extracellular vesicles derived from MSCs can restore TRPC6 channel activity and mitochondrial functions, correct calcium homeostasis, and significantly promote healing in diabetic wounds^272^.

Furthermore, innovative delivery systems are also emerging. For instance, Dong *et al.* developed functional mitochondria-loaded microvesicles (Mito@euMVs) from enucleated MSCs using a simple extrusion process. Transdermal delivery of these Mito@euMVs via microneedle patches to diabetic rats with pressure sores effectively inhibited and reversed hyperglycemia-induced cellular senescence, thereby facilitating chronic wound healing^273^. Likewise, Shan He *et al.* created a biomimetic gene delivery platform combining VEGF-loaded, extracellular vesicle-encapsulated adeno-associated virus (AAV) with human umbilical cord MSC-derived exosomes embedded in a hydrogel. Application of this system to diabetic wound models improved mitochondrial function in endothelial cells, enhanced vascularization, and promoted chronic wound healing by modulating the inflammatory microenvironment^274^. Collectively, these advances underscore the therapeutic potential of mitochondria transfer, whether via direct mitochondrial donation, engineered vesicles, or advanced delivery systems, in the treatment of chronic and acute wounds. As our understanding of mitochondrial dynamics and intercellular communication deepens, mitochondria transfer is poised to become a transformative strategy in regenerative medicine.

#### Respiratory injury and inflammation

Mitochondria transfer is a critical therapeutic tool for respiratory injuries and inflammatory conditions, such as acute lung injury (ALI) and chronic obstructive pulmonary disease (COPD). Mitochondrial complex I-dependent NAD^+^ regeneration, potentially influenced by mitochondria transfer, is essential for proper lung epithelial cell fate during postnatal alveolar development by preventing pathological activation of the integrated stress response^275^. In ALI, MSCs transfer mitochondria to damaged alveolar epithelial cells via TNTs or EVs, restoring ATP production, reducing inflammation, and repairing the epithelial barrier^107^, ^147^. Notably, BMSCs airway-instilled into mice with LPS-induced acute lung injury formed Cx43-dependent GJs with alveolar epithelial cells and transferred mitochondria via microvesicles, thereby increasing alveolar ATP levels and significantly reducing lung injury and mortality^107^. In COPD, mitochondria transfer from MSCs or macrophages protects lung cells against oxidative stress-induced mitochondrial dysfunction and apoptosis and reduces pro-inflammatory cytokine release^276^, ^277^. Beyond cell-based approaches, pharmacological modulation of mitochondrial function has demonstrated substantial promise for improving local inflammation and tissue repair. For instance, in mouse models of ALI and viral pneumonia, nanovesicles derived from the medicinal plant *Artemisia annua* have been used to deliver gamma-aminobutyric acid (GABA) to alveolar macrophages, thereby enhancing mitochondrial bioenergetics, dampening lung inflammation, and improving survival outcomes^278^. These findings suggest that mitochondria transfer could transform the management of acute and chronic respiratory diseases.

#### Central nervous system (CNS) injury

Mitochondrial dysfunction has been identified as a seemingly unifying pathological phenomenon across a wide range of neurodegenerative disorders. Recent studies have shown that mitochondrial transplantation has been demonstrated to replace damaged or dysfunctional mitochondria with exogenous healthy mitochondria for CNS injuries, including stroke, traumatic brain injury (TBI), and spinal cord injury (SCI). The transfer of healthy mitochondria into injured cells has been shown to restore ATP levels and reduce energy deficits following ischemic stroke^279^. Stem cell-based mitochondria transfer thus represents a promising therapeutic strategy for stroke^280^. After ischemia-reperfusion injury, MSCs maintain aerobic respiration and inhibit apoptosis in damaged endothelial cells through mitochondria transfer^280^. Several preclinical and clinical studies have evaluated mitochondria-targeted therapies, indicating that stem cell-based treatments and mitochondrial transplantation are promising novel biotherapeutic strategies for the treatment of traumatic brain injury (TBI)^281^-^283^. In SCI, mitochondria transfer from MSCs to damaged neurons supports axonal regeneration and functional recovery^284^-^286^. These approaches are complemented by cell engineering strategies, enhancing delivery efficiency, offering hope for CNS repair.

#### Other diseases

In addition to the common diseases mentioned above, mitochondria transfer holds significant potential for the treatment of various other pathological conditions and the maintenance of systemic homeostasis. Alway *et al.* demonstrate that systemic mitochondria delivery can enhance the rate of muscle regeneration and recovery of muscle function after injury^287^. Moreover, in a collagenase-induced mouse model of osteoarthritis, intra-articular injection of MSC-derived mitochondria protects against cartilage degeneration by restoring chondrocyte energy metabolism and mitochondrial dynamics, while enhancing resistance to oxidative stress and apoptosis^288^. In a study focused on the autoimmune tissue inflammation of rheumatoid arthritis, Wu B *et al.* reported that transfer intact mitochondria into T cells, as well as supplementation of exogenous aspartate, rescued the mitochondria-instructed expansion of ER membranes and suppressed TNF release and rheumatoid tissue inflammation^289^. Similarly, the transfer of mitochondria to BMSCs, followed by in situ implantation at cranial defect sites, has been demonstrated to significantly promote bone defect repair^290^. Moreover, published study demonstrates that MSCs deliver hypoxia-conditioned functional mitochondria to injured pancreatic acinar cells via EVs, reprogramming their metabolism and alleviating severe acute pancreatitis injury^291^. Wei *et al.* demonstrates that BMMSCs protect against tendinopathy by transferring healthy mitochondria to injured tenocytes both *in vitro* and *in vivo*, thereby restoring mitochondrial functions and promoting tendon healing^292^.

### Current clinical trails

To further review the current clinical applications and outcomes in this field, registered clinical trials and published first-in-human or pilot clinical experience related to mitochondrial transplantation and mitochondria transfer-based therapies were examined and evaluated (***Table 3***). During data searching and literature review, we found that mitochondria-based interventions have been registered in ClinicalTrials.gov across multiple contexts, including acute ischemic stroke^293^, cardiac ischemia or extracorporeal membrane oxygenation (ECMO)-associated myocardial injury (NCT02851758), and inflammatory myopathies (NCT04976140). Moreover, autologous mitochondrial transplantation is currently being evaluated in a registered clinical study for cerebral ischemia (NCT04998357), sponsored by the University of Washington. This ongoing trial further indicates that mitochondrial transplantation has progressed beyond preclinical investigation and is under active clinical evaluation. In addition, a pediatric postcardiotomy cardiogenic shock or ECMO setting has published pilot outcomes associated with autologous mitochondrial transplantation approaches^294^. Collectively, these records support that mitochondria transplantation and transfer-based therapies have entered early clinical exploration, while the current evidence base remains predominantly early-phase, small sample, and frequently non-randomized, and therefore efficacy conclusions remain premature.

## Current progress in HMT methodology

With growing attentions and great efforts on the fundamental studies and therapeutic trails of mitochondria transfer, different applications regarding the improvement of transferring efficiency by engineered cell therapies, various transplantation strategies, and pharmacological agents are currently enhancing our understanding of mitochondria transfer and their potential as a treatment for different diseases.

### Mitochondrial transplantation

Direct transplantation of isolated, functional mitochondria into damaged tissues has been explored in a range of animal models and early clinical studies, revealing its potential for restoring tissue function. Recent findings indicate that specific sub-populations of mitochondrial vesicles (mitoEVs), which are enriched in mitochondrial components, can effectively deliver these elements to recipient cells and modulate their function under various pathological conditions^295^. The concept of HMT has thus inspired the development of innovative mitochondrial transplantation strategies for human disease. Emerging technologies are rapidly expanding the toolkit for mitochondrial transplantation. Artificial mitochondria generated through mitochondrial genome engineering or mitochondrial-nuclear hybridization offer new avenues for treating diseases associated with mitochondrial dysfunction^296^. Notably, Gäbelein *et al.* developed a dedicated probe based on the FluidFM platform that can minimally invasively access living cells to extract, inject, and transplant mitochondria with subcellular spatial resolution; importantly, the transplanted mitochondria can integrate into the host cell's mitochondrial network and support replication of donor mitochondrial DNA. Recently, Hyslop, L. A., *et al.* demonstrated that mitochondrial donation through pronuclear transfer was compatible with human embryo viability, which was effective in reducing the transmission of homoplasmic and heteroplasmic pathogenic mtDNA variants^297^. In addition, Kim *et al.* developed a novel and efficient therapeutic platform consisting of fusogenic liposomes encapsulating mitochondria, which showed promising results in delivering mitochondrial proteins and was validated in the treatment of osteoarthritis^298^. These advanced approaches are opening new perspectives for the study and treatment of mitochondrial diseases, paving the way for innovative clinical applications.

### Cell engineering using extracellular mitochondria

Engineered cell therapies using extracellular mitochondria represent a new frontier. Restoring the mitochondrial health of cells with mitochondrial dysfunction using embryonic stem cells, MSCs, iPSCls, stem cell secretions, and EV-mediated mitochondrial transplantation technology can affect important processes such as their proliferation, differentiation, cell metabolism, inflammatory response, cell senescence, cell stress, and cell migration, and enhance their regenerative capacity, survival rate, and therapeutic effect, which is crucial to improving therapeutic effects^299^. It holds great therapeutic potential for the treatment of neurological disorders, tissue repair, lung injury, tumors, and other diseases. Recent evidence showed that the release of haematopoietic cell-derived extracellular mitochondria into circulation, or direct transfer isolated healthy mitochondria from mice or human can extend lifespan, improve neurological function in Leigh syndrome (LS) mouse models^300^. Notably, accumulating evidence indicates that mitochondrial transfer from MSCs plays a dual role in promoting both tissue repair and cancer progression, highlighting its potential as a cell engineering strategy for regenerative medicine and cancer therapy^301^, ^302^.

### Drugs and nanomedicine affecting mitochondria transfer

Beyond their well-established role in energy metabolism, mitochondria are now appreciated as integrative hubs where catabolic and anabolic pathways, redox mediators, and diverse signaling networks intersect to sustain cellular homeostasis and orchestrate responses to environmental cues. Thus, understanding how pharmacological agents affect mitochondrial biochemistry and mitochondria transfer is therefore of critical importance and holds substantial promise for the development of innovative therapies targeting mitochondrial-related diseases^303^. Numerous studies have shown that drugs that can modulate mitochondrial transport are under development. Small molecules that promote TNT formation, actin polymerization, or mitochondrial transport can promote beneficial mitochondrial transport in regenerative environments, while TNT inhibitors or vesicle transport blockers may limit pathological metastasis in cancer^304^. Besides pharmaceutic drugs, new materials, especially nanoparticles, have been widely tested and engineered to enhance mitochondria transfer *in vitro* and *in vivo*. For instance, Huang T *et al.* reported that iron oxide nanoparticles (IONPs) can augment the intercellular mitochondrial transfer from hMSCs selectively to diseased cells through enhanced Cx43- mediated GJs^305^.

## Conclusions and perspectives

For the past two decades, HMT has emerged as a new paradigm in cell biology occurring *in vitro* and *in vivo*. More distinct transferring modes have been reported between cells in various tissue background. However, the remaining fundamental questions regarding HMT include: (1) What is the precise onset and triggering information for HMT? (2) What are the molecular players and orchestrated sequences of connection formation and transportation? (3) How to apply the therapeutic potential of HMT in clinic?

During the initiation of HMT, one crucial question is yet to be answered is the trigger of intercellular connections, or the trigger of Ev-Mito or Ex-Mito formation. Since HMT is commonly reported between healthy and stressed cells, one can hypothesize that the potential “signal” triggering the process of HMT arise from the receptor cells, which are in need of functional mitochondria^306^. Current published data showed that the triggering signal is presented by ADP in the stress osteocytes^306^. From the fundamental cell biology, this initiation information is intriguing and could be harnessed for alleviating various pathological states. During HMT formation and mitochondria transportation, directly visualization of mitochondria transfer would unreal its physiological significance. Current studies utilized fluorescence dyes to track mitochondria dynamics. However, the leakage of dyes results in limitations or even false results. The development of novel fluoresceins for mitochondrial tracking and visualizing HMT in real time *in vivo* remain major challenges in this field. Moreover, separating healthy and damaged mitochondria with different labeling during HMT could be another challenging which could greatly accelerate the progress.

In summary, considering the broad involvement of mitochondria transfer with implications for tissue homeostasis, physiology, and pathophysiology, future therapeutic implications need to enhance desirable transfer, bock non-wanted transfer and achieve functional treatment in receptor cells and tissues. In a broad point of view, segregation of mitochondria derived from various origins may bring new insights into “inter-organ mitohormesis”^127^, ^307^. These distant mitochondrial communications involved in multiple organs or even entire organism could explain the pathological mechanisms behind some systematic diseases, such as diabetes, stress, aging, lifespan and etc. From this perspective, mitochondria transplantation or mitochondria-related treatment may bring new therapeutics with high translational potential for treating diseases that are linked to mitochondrial dysfunctions.

## Figures and Tables

**Table 1:** Mitochondria transfer reported in different tissue backgrounds.

Tissues/Organs	Donor cells (Cell type/species)	Recipient cells (Cell type/species)	Mode of MT transfer	Functions	Ref.
Immune systems	MSC/Human	Alveolar macrophages/Murine	EVs	Ameliorate lung injury *in vivo*	148
	M1-like macrophage/Mouse	MSCs/Mouse	TNTs and EVs	Induce ROS bursts, impair the osteogenic differentiation of MSCs	253
	Breast cancer cells/Human	T-cells/Human	TNTs	Lead to T cell exhaustion	246
	T cells/Mouse	Breast cancer cells/Human, Breast cancer cells/Mouse	TNTs	Facilitate immune evasion	153
Nervous system	M2-like macrophage/Mouse	Sensory neurons/Mouse	EVs	Reduce inflammatory pain	152
	BMSCs/Rat	Cortical neurons/Rat	GJs	Protect neural cells from apoptosis	103
	Microglia/Mouse	Neurons/Mouse	TNTs	Restore neuronal health	308
	Astrocytes/Human	Glioblastoma/Human	Intercellular transfer through network-forming	Upregulate metabolic pathways related to proliferation and tumorigenicity	309
	Astrocytes/Mouse	Neurons/Rat	TNTs	Support neuronal mitochondrial metabolism and survival	119
Cardiovascular system	Cardiomyoblast cells/Rat	H9c2 cardiomyocytes/Mouse	Ex-Mito	Alleviates myocardial ischemia-reperfusion injury	183
	M2-like macrophage/Mouse	H9c2 cardiomyocytes/Rat	Cell fusion	Prevent doxorubicin-induced cardiotoxicity	310
	Colon carcinoma cell line/Human	Neonatal cardiomyocytes/Mouse	Ex-Mito	Protect cardiomyocytes from doxorubicin-induced mitochondrial dysfunction	181
	hUC-MSC/Human	Cardiomyocytes/Mouse	Ex-Mito	Repair myocardial injury	182
	Osteocytes/Mouse	Endothelial cells/Mouse	Ex-Mito	Regulate the vascularization of transcortical vessels	118
	MSCs/Human	Endothelial cells/Human	TNTs	Promote EC transplantation and stimulating angiogenesis	184
Respiratory system	MSCs/Human	Bronchial epithelial cells/Human	TNTs	Mediate the repair of damaged bronchial epithelial cells	186
	Asthmatics' airway myeloid-derived regulatory cell/Human	CD4+ T/Human	EVs	Reprogramme the function of T cells and inhibite their inflammatory responses	187
	Airway smooth muscle cells (ASMCs)/Human	Airway smooth muscle cells (ASMCs)/Human	EVs	Regulate bioenergetics and cellular functions within the airway	189
	hP-MSC/Human	TC-1 epithelial/Mouse, HUVEC endothelial/Human, Hs888Lu fibroblast/Human	Ex-Mito	Repair the damaged mitochondria, restore the inhibited mitochondrial autophagy process	191
	BMSCs/Mouse	Pulmonary microvascular endothelial cells/Mouse	TNTs	Restore the function of mitochondria, reduce the levels of ROS and cell apoptosis, recovery the barrier function of ECs	192
Liver	hUC- MSCs/Human	AML12 liver/Mouse	TNTs	Restored mitochondrial function, activate the Nrf2/HO-1 signaling pathway in hepatocytes, suppress ferroptosis and fibrosis	199
	hUC-MSCs/Human	Neutrophils/Murine	EVs	regulate neutrophil NETs formation	200
	BMMSCs/Human	Primary hepatocytes/Mouse	TNTs	Enhance lipid metabolism and facilitate the restoration of tissue homeostasis	197
	MIHA cells (Immortalized hepatocytes)/Human	NaAsO2-treated MIHA cells	TNTs	Enable the mutual supplementation of mtDNA between cells, compensating for the mitochondrial dysfunction caused by arsenic and alleviates cellular senescence	198
	PLC/PRF/5 cells/Human, MHCC-97H cells/Human	Hep3B cells/Human	TNTs	Enhance the migration and invasion abilities of the less invasive cells	201
Kidney	BMSCs/Rat	Glomerular endothelial cells/Rat	Contact-independent Mt transfer( co-culture, the specific mode was not explicitly stated. )	Anti-apoptotic	210
	BMSCs/Rat	Proximal tubular epithelial cells/Rats	Contact-dependent Mt transfer (co-culture, the specific mode was not explicitly stated. )	Inhibit cell apoptosis, inhibit ROS production, restore transporter expression, and repair renal tubular structure	311
Musculoskeletal system	MLO-Y4 osteocyte/Mouse	MLO-Y4 osteocyte/Mouse	Dendiritic network	Restore the metabolic function of injured mitochondria and maintain the homeostasis of bone cells	116
	RAW 264.7 macrophage/Mouse	BMSCs/Mouse	TNTs, EVs	Promote the osteogenic differentiation of MSCs and regulate the homeostasis of bone.	253
	BMSCs/Mouse	Chondrocytes/Mouse	TNTs, GJs	Improve the mitochondrial function of chondrocytes	312
	BMSCs/Rat	Chondrocytes/Rat	Contact-dependent Mt transfer (co-culture, the specific mode was not explicitly stated. )	Improve mitochondrial function, enhance cell proliferation, and inhibit apoptosis	217
Adipose tissues	Adipocytes/Mouse	Macrophages/Mouse	Not well understood	Regulate metabolic homeostasis	221
	Adipocytes/Mouse	Cardiomyocytes/Mouse	EVs	Protect cardiomyocytes from acute oxidative stress	127
	ADSCs/Rat	Dendritic cells/Rat	EVs	Reduce inflammation	224
	ADSCs/Mouse	Macrophages/Mouse	TNTs	Promote barrier restoration	223
	Macrophages/Mouse	Adipocytes/mouse	EVs	Promote Adipocyte-myofibroblast transition	222
	ADSCs/Human	Cardiomyocytes/Rat	TNTs	Improve the cardiac function	226
	ADSCs/Human	islet β-cells human	TNTs and EVs	Improve insulin secretory function	225
	ADSCs/Human	Breast cancer cells/Human	TNTs	multi-drug resistance (MDR)	227
Reproductive system	ADSCs/Mouse	Oocytes/Mouse	microinjection	Enhance oocyte quality, promote embryonic development	228
	EnMSCs/Mouse	Oocytes/Mouse	microinjection	Enhance oocyte quality, promote embryonic development	229
	iPSCs/Mouse	Oocytes/Mouse	microinjection	Enhance oocyte quality, promote embryonic development	230
	USCs/Human	Oocytes/Mouse	microinjection	Improve embryonic development and metabolism	231
	ASCs/Mouse	Oocytes/Mouse	microinjection	Improve the developmental potential of cryopreserved oocytes	232

**Table 2:** Therapeutic impact of mitochondria transfer in pathological conditions.

Pathological conditions	Disease/ injury models	Mitochondria/ Treatment	Mode/Route of transfer	Outcomes	Ref.
Inherited mitochondrial diseases	Friedreich ataxia, LHON, MELAS, MERRF, Leigh syndrome, mtDNA depletion syndromes, MNGIE	Mitochondrial transplantation/exogenous mitochondrial delivery Supplementation (CoQ10, idebenone, EPI-743) High-dose L-arginine (MELAS) Allogeneic stem cell transplantation, enzyme replacement (MNGIE) Deoxynucleoside supplementation (mtDNA depletion) Rapamycin (autophagy/mitophagy modulation) NAD⁺ enhancement (nicotinamide riboside)	Mitochondrial transplantation, exogenous mitochondrial delivery Oral or intravenous drug/supplement therapy Cell transplantation	Improved mitochondrial function and metabolism Reduced stroke-like episodes (MELAS) Improved survival and quality of life Correction of underlying defects in preclinical models	255-261
Cancers	Tumor/cancer models (including preclinical, T cell/CAR-T cell models)	MSC/fibroblast-derived mitochondria TNT inhibitors (e.g., vincristine), selective mitochondrial depletion TrxR2 inhibition MSC mitochondrial transfer to Treg or CAR-T cells	TNTs, EVs Pharmacological/genetic blockade Pharmacological inhibition HLA-dependent cell contact	Restored mitochondrial respiration Reduced tumor growth, increased therapy sensitivity Promoted cancer cell apoptosis Enhanced immunosuppressive function of Tregs	263-267
Wound healing	Diabetic wound models, diabetic rats with pressure sores, full-thickness skin wounds in mice, cranial defect models	MSC-derived EVs and small EVs carrying mitochondria HucMSC-derived exosomes (HucMSC-Exo) Mitochondria-loaded microvesicles (Mito@euMVs) VEGF-loaded AAV encapsulated in HucMSC-exosome hydrogel Engineered/optimized MSCs for mitochondrial transfer	EV-mediated or exosome injection Microneedle transdermal delivery Hydrogel-based gene delivery Direct cell engineering/local transfer	Restored neutrophil and endothelial mitochondrial function Suppressed NETs and ferroptosis Restored calcium homeostasis Inhibited cell senescence Enhanced angiogenesis and vascularization Accelerated wound/bone defect healing and tissue regeneration	269-274, 290
Respiratory system: injury and inflammation	ALI (e.g., LPS-induced), COPD models, viral pneumonia in mice, postnatal alveolar development	MSC- or macrophage-derived mitochondria Mitochondrial complex I-dependent NAD⁺ regeneration Plant nanovesicles (Artemisia annua) delivering GABA	TNTs, EVs, microvesicles Nanovesicle-mediated delivery Cell-based or pharmacological modulation	Restored ATP and mitochondrial bioenergetics Reduced inflammation and oxidative stress Repaired epithelial barrier Improved survival and cell fate Decreased cytokine release and apoptosis	107, 147, 275-278
Central nervous system (CNS) injury	Ischemic stroke, traumatic brain injury (TBI), spinal cord injury (SCI)	Exogenous healthy mitochondria transplantation MSC-based mitochondrial transfer Mitochondria-targeted therapies Cell engineering approaches	Direct mitochondrial transplantation Stem cell-mediated mitochondrial transfer Cell engineering to enhance delivery	Restored ATP levels and aerobic respiration Reduced energy deficits and apoptosis Supported axonal regeneration and functional recovery Improved therapeutic outcomes	279-286
Other diseases	1.Muscle injury;2. OA chondrocytes (*in vitro*), collagenase-induced OA in mice;3. Injured pancreatic acinar cells (severe acute pancreatitis); 4.Tendinopathy (*in vitro* & *in vivo*)	Systemic mitochondrial delivery MSC-derived mitochondria/microtubules Hypoxia-conditioned MSC-derived mitochondria Bone marrow MSC-derived healthy mitochondria	Systemic administration Intra-articular injection Extracellular vesicles (EVs) Direct transfer	Enhanced muscle regeneration and function Restored energy status & mitochondrial dynamics increased resistance to oxidative stress & apoptosis Protected against cartilage degeneration Reprogrammed cell metabolism, alleviated pancreatitis injury Restored mitochondrial function, promoted tendon healing	287, 288, 291, 292

**Table 3:** Current mitochondria-based therapeutic treatments in clinical trails.

Diseases	Mt sources and approach	Sample size	Phase / status	Outcomes	Clinical trial#
Acute ischemic stroke	Autologous mitochondria;intra-arterial/endovascular delivery to ischemic territory.	Early-phase feasibility	Phase 1; recruiting	No major safety signal reported in Phase 1 publication.	Clinical trial NCT04998357 293
Pediatric cardiac ischemia	Autologous mitochondria; direct injection or infusion to myocardium.	Registry lists pediatric ECMO; publication pilot cohort	Phase NA; unknown	Pilot outcomes reported in pediatric cardiogenic shock/ECMO context.	Clinical trial NCT02851758 294
Refractory polymyositis	Allogeneic mitochondria product; systemic administration.	Dose-escalation small cohorts described in registry snippet	Phase 1/2a; recruiting	Safety/tolerability and exploratory efficacy.	Clinical trial NCT04976140
Refractory polymyositis (later phase)	Allogeneic mitochondria product; systemic administration.	Not specified	Phase 2; recruiting	Designed to evaluate efficacy/safety.	Clinical trial NCT07122648
Cardiac ischemia	Autologous mitochondria with MSC-exosomes (co-therapy)	Not specified	Phase 1/2; unknown	Study designed to evaluate safety, feasibility, and preliminary efficacy; no results published or posted yet.	Clinical trial NCT05669144
Pearson syndrome	Autologous CD34^+^ cells with donor-derived mitochondria.	7 participants	Phase 1/2; Completed.	Trial completed; no peer-reviewed publication of outcomes.	Clinical trial NCT03384420
Reproductive medicine	Autologous mitochondria transfer to oocyte.	Pilot	Pilot; unknown	Designed to assess feasibility of improving oocyte quality by autologous mitochondrial transfer; no published results available.	Clinical trial NCT03639506
